# Functional and Structural Characterization of Diverse NfsB Chloramphenicol Reductase Enzymes from Human Pathogens

**DOI:** 10.1128/spectrum.00139-22

**Published:** 2022-02-23

**Authors:** Michael W. Mullowney, Natalia I. Maltseva, Michael Endres, Youngchang Kim, Andrzej Joachimiak, Terence S. Crofts

**Affiliations:** a Department of Chemistry, Northwestern University, Evanston, Illinois, USA; b Center for Structural Genomics of Infectious Diseases, Consortium for Advanced Science and Engineering, University of Chicago, Chicago, Illinois, USA; c Structure Biology Center, Argonne National Laboratory, Argonne, Illinois, USA; d Department of Biochemistry and Molecular Biology, University of Chicago, Chicago, Illinois, USA; e Department of Molecular Biosciences, Northwestern University, Evanston, Illinois, USA; University of Pittsburgh

**Keywords:** antibiotic resistance, chloramphenicol, crystal structure, *Haemophilus influenzae*, *Neisseria*, NfsB, nitroreductase

## Abstract

Phylogenetically diverse bacteria can carry out chloramphenicol reduction, but only a single enzyme has been described that efficiently catalyzes this reaction, the NfsB nitroreductase from Haemophilus influenzae strain KW20. Here, we tested the hypothesis that some NfsB homologs function as housekeeping enzymes with the potential to become chloramphenicol resistance enzymes. We found that expression of H. influenzae and *Neisseria* spp. *nfsB* genes, but not Pasteurella multocida *nfsB*, allows Escherichia coli to resist chloramphenicol by nitroreduction. Mass spectrometric analysis confirmed that purified H. influenzae and N. meningitides NfsB enzymes reduce chloramphenicol to amino-chloramphenicol, while kinetics analyses supported the hypothesis that chloramphenicol reduction is a secondary activity. We combined these findings with atomic resolution structures of multiple chloramphenicol-reducing NfsB enzymes to identify potential key substrate-binding pocket residues. Our work expands the chloramphenicol reductase family and provides mechanistic insights into how a housekeeping enzyme might confer antibiotic resistance.

**IMPORTANCE** The question of how new enzyme activities evolve is of great biological interest and, in the context of antibiotic resistance, of great medical importance. Here, we have tested the hypothesis that new antibiotic resistance mechanisms may evolve from promiscuous housekeeping enzymes that have antibiotic modification side activities. Previous work identified a Haemophilus influenzae nitroreductase housekeeping enzyme that has the ability to give Escherichia coli resistance to the antibiotic chloramphenicol by nitroreduction. Herein, we extend this work to enzymes from other Haemophilus and *Neisseria* strains to discover that expression of chloramphenicol reductases is sufficient to confer chloramphenicol resistance to Es. coli, confirming that chloramphenicol reductase activity is widespread across this nitroreductase family. By solving the high-resolution crystal structures of active chloramphenicol reductases, we identified residues important for this activity. Our work supports the hypothesis that housekeeping proteins possessing multiple activities can evolve into antibiotic resistance enzymes.

## INTRODUCTION

Rates of infection by antibiotic-resistant bacteria are steadily increasing. Annual deaths due to previously curable bacterial infections in the United States are estimated between 23,000 and greater than 150,000 and are predicted to reach 10 million globally by the year 2050 (1–3). Among the most concerning antibiotic-resistant bacteria are those carrying newly evolved resistance mechanisms (4–6). Combatting the evolution of novel resistance mechanisms requires a thorough understanding of the evolutionary paths taken by these enzymes. Multiple routes for the evolution of antibiotic-modifying enzymes from central metabolism or “housekeeping” enzymes have been hypothesized (4). In this context, housekeeping enzymes that modify xenobiotics are generally characterized by slow kinetics, broad substrate utilization, genomic context, and often noninduced expression (4). One of the best examples of a housekeeping gene that evolved into an antibiotic resistance gene is the β-lactamases, which are thought to have originated as transpeptidase targets of β-lactam antibiotics (4, 6). The ability to study a housekeeping gene with the potential to evolve into a bona fide resistance gene would provide a highly useful model in the fight against antimicrobial resistance.

**FIG 1:**
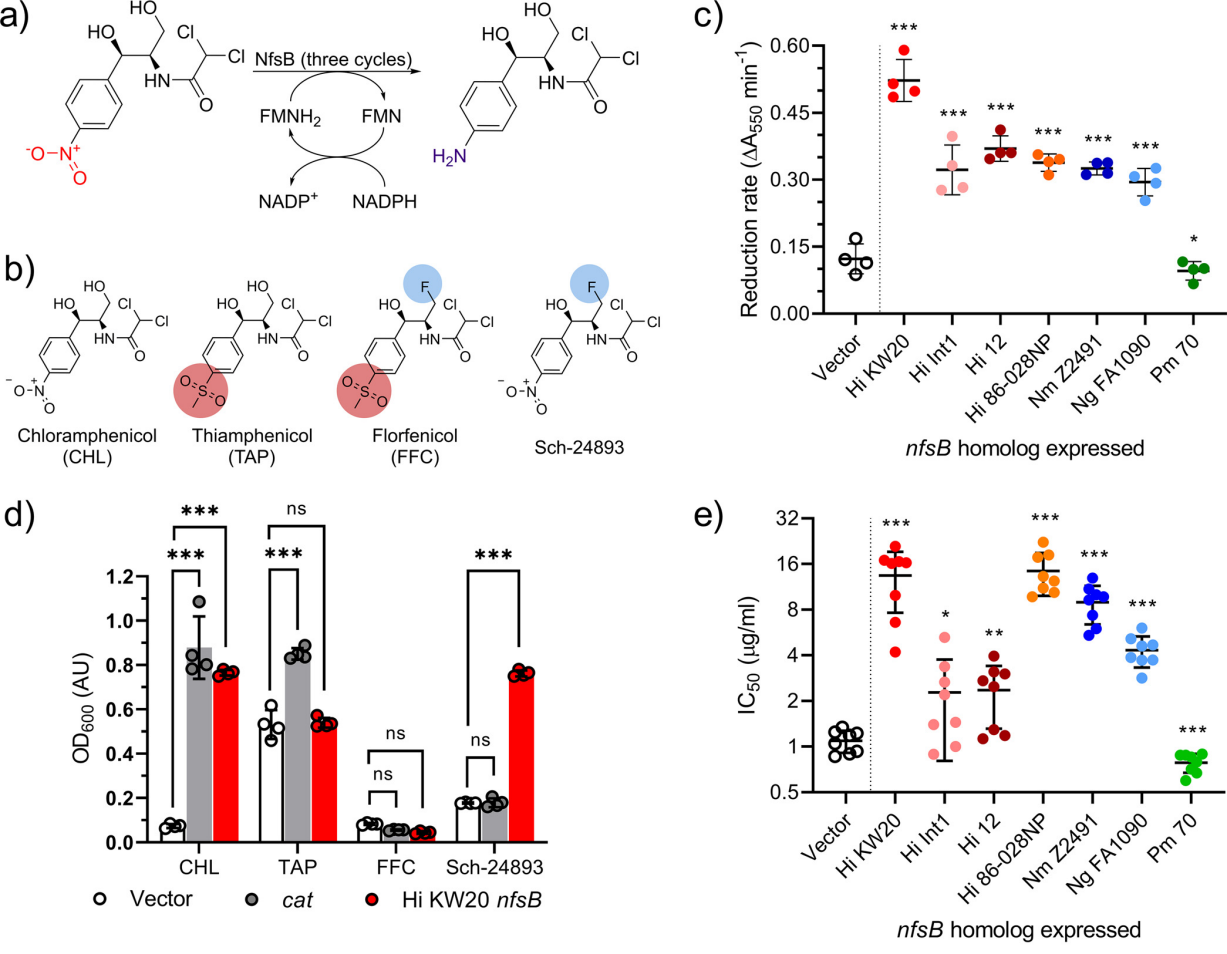
Chloramphenicol reduction and resistance in Es. coli expressing *nfsB* homologs. (a) Enzymatic or cellular reduction of the chloramphenicol nitro group to form amino-chloramphenicol. NADPH serves to reduce the NfsB flavin mononucleotide (FMN) prosthetic group, which in turn reduces the nitro substrate. Complete reduction requires three enzymatic cycles. (b) Chemical structures of chloramphenicol and its derivative antibiotics with key structural changes highlighted: replacement of the *p*-nitro group with a *p-*methyl-sulfonyl group (red) and/or replacement of a hydroxyl group with a fluorine (blue). (c) Chloramphenicol reduction rates of Es. coli cultures expressing the indicated *nfsB* gene homologs, as detected by Bratton-Marshall derivatization. Biological replicates with mean and standard deviation error bars (*n* = 4) are shown. Reduction rates were compared to vector control, and significance was determined by Brown-Forsythe and Welch ANOVA tests with Benjamini, Krieger, and Yekutieli false-discovery rate correction; A_550_, absorbance at 550 nm; *, *q* < 0.05; **, *q* < 0.005; ***, *q* < 0.0005. (d) Optical density at 600 nm (OD_600_) of Es. coli cultures expressing an empty vector (vector), chloramphenicol acetyltransferase (*cat*), or the H. influenzae KW20 *nfsB* (Hi KW20 *nfsB*) following overnight growth in the presence of the indicated amphenicol at 4 µg/mL. Biological replicates (*n* = 4) with mean and standard deviation error bars are shown. Statistical testing was performed as in panel c above; ns, not significant. (e) Chloramphenicol 50% inhibitory concentration (IC_50_) values for Es. coli strains expressing the indicated *nfsB* homolog. Shown are means and standard deviation error bars of biological replicates (*n* = 8). Significance was determined by comparison to the vector-only control as in panel c above. Abbreviations: Hi, Haemophilus influenzae; Nm, Neisseria meningitidis; Ng, Neisseria gonorrhoeae; Pm, Pasteurella multocida; CHL, chloramphenicol; TAP, thiamphenicol; FFC, florfenicol.

Here, we propose NfsB nitroreductase enzymes and their homologs as models for housekeeping enzymes with antibiotic resistance activity, in this case against the antibiotic chloramphenicol. While the most commonly encountered mechanism of bacterial resistance to chloramphenicol is by acetylation (7), the earliest recorded mechanism was by reduction of the nitro moiety (8) (Fig. 1a). Reduction of chloramphenicol to amino-chloramphenicol has been reported in a variety of bacterial strains (9–12) and has contributed to treatment failure in an animal model that used this antibiotic (13). Reduction of the chloramphenicol nitro group has also been hypothesized to underlie the serious chloramphenicol side effect of aplastic anemia, and prolonged exposure has also been associated with renal toxicity (14). Recently, we found that a Haemophilus influenzae homolog of the NfsB nitroreductase enzyme can confer clinical levels of antibiotic resistance when expressed in Escherichia coli (15). We and others have demonstrated that this enzyme, specifically from H. influenzae strain Rd KW20, can reduce the nitro group of chloramphenicol to the corresponding amine *in vitro* (15–17). It is via this promiscuous nitro reductase activity that NfsB confers chloramphenicol resistance.

Previous studies have used saturating mutagenesis of experimenter-determined sites alone (18) or in combination with ancestral reconstruction (19) to attempt to evolve the specificity of enzyme-antibiotic interactions with various degrees of success. Here, we have taken advantage of a “natural experiment” provided by divergent evolution, resulting in proteobacterial NfsB homologs with reciprocal amino acid identities ranging from 57.27% to 99.09% (Table S1 in the supplemental material). These allow us to examine the effects of naturally occurring mutations on chloramphenicol reduction and correlate them with resistance. We couple this information to high-resolution structural data to infer the relative importance of specific residues for chloramphenicol modification.

## RESULTS

### Selection of *nfsB* homologs for cloning into Es. coli.

Previous work has demonstrated that the NfsB enzyme from H. influenzae strain Rd KW20 (Hi KW20) has the ability to reduce chloramphenicol to amino-chloramphenicol *in vitro* (15–17). Smith et al. initially identified several Haemophilus strains, including Hi KW20, and taxonomically unrelated bacterial strains as having the ability to reduce chloramphenicol in bacterial culture (12). In order to test if chloramphenicol reduction in these strains is also mediated by NfsB enzymes, we selected NfsB homologs from six additional bacteria for investigation alongside Hi KW20 NfsB: H. influenzae strain Int1 (Hi Int1), H. influenzae strain 12 (Hi 12), H. influenzae strain 86-028NP (Hi 86-028NP), Neisseria gonorrhoeae strain FA1090 (Ng FA1090), N. meningitidis strain Z2491 (Nm Z2491), and Pasteurella multocida strain 70 (Pm 70). These NfsB homologs included enzymes predicted to reduce (Hi KW20, Hi Int1, Hi 12, Hi 86-028NP, Ng FA1090, and Nm Z2491) or not reduce (Pm 70) chloramphenicol based on the phenotypes detailed in Smith et al. (12). The homologs also showed a range of evolutionary relatedness (inferred by amino acid sequence identity) to Hi KW20 NfsB. The four NfsB homologs from H. influenzae strains show a high degree of relatedness as measured by overall sequence identity at the amino acid level (94.98% to 99.09%). The two *Neisseria*-derived NfsB enzymes show high amino acid identity (96.83%) to each other but only moderate identity to the H. influenzae homologs (56.82% to 59.09%). Finally, the P. multocida NfsB protein shares between 58.64% and 61.19% amino acid sequence identity to the other homologs (Table S1 in the supplemental material) despite being evolutionarily more closely related to H. influenzae (shared family *Pasteurellaceae*) than *Neisseria* sp. (shared phylum of *Proteobacteria*).

### Reduction of the chloramphenicol nitro moiety by Es. coli expressing heterologous *nfsB* genes.

To test the hypothesis that NfsB activity underlies phenotypic chloramphenicol reduction, we assayed cultures of Es. coli expressing each *nfsB* homolog identified above for reduction of the chloramphenicol nitro group to an amine. Dense cultures of each Es. coli *nfsB-*expressing strain were washed and incubated with chloramphenicol to initiate reduction of the nitro group (Fig. 1a) (10). Production of aromatic amino groups (such as in amino-chloramphenicol) was quantified by Bratton-Marshall assay (12, 15, 20) to determine the reduction rates of the cultures. Cell-free supernatants from the empty vector control and Es. coli strain expressing the Pm 70 *nfsB* gene reactions showed negligible derivatization by the Bratton-Marshall reagent, while supernatants from the Es. coli strains expressing H. influenzae or *Neisseria nfsB* genes showed robust signal (Fig. 1c), with the strain expressing the Hi KW20 *nfsB* showing the highest apparent reduction rate.

### NfsB-mediated chloramphenicol resistance in Es. coli.

Next, to further link resistance to nitro reduction, we challenged vector control Es. coli and Es. coli expressing a chloramphenicol acetyltransferase (*cat*) gene or the Hi KW20 *nfsB* gene by cultivation at inhibitory concentrations of amphenicol antibiotics. These antibiotics included chloramphenicol (vulnerable to both resistance mechanisms), thiamphenicol (theoretically vulnerable to *cat* acetylation but invulnerable to *nfsB* nitroreduction due to replacement of the nitro group with a methyl-sulfonyl group), florfenicol (theoretically invulnerable to both genes), and the amphenicol analog Sch-24893 (theoretically invulnerable to *cat* but vulnerable to *nfsB*) (Fig. 1b). Expression of either *cat* or *nfsB* was sufficient to confer resistance to chloramphenicol, while neither resistance mechanism allowed for growth in the presence of florfenicol. In the presence of thiamphenicol, *cat* expression conferred resistance, while *nfsB* expression did not. Conversely, *cat* expression was not sufficient to allow Es. coli to grow in the presence of Sch-24893, while *nfsB* expression was sufficient (Fig. 1d).

Finally, we measured the chloramphenicol susceptibility of each *nfsB*-expressing Es. coli strain compared to a vector control. Concentration-response curves of overnight growth measured against various concentrations of chloramphenicol were generated (Fig. S2A and B) and used to calculate the chloramphenicol 50% inhibitory concentration (IC_50_) for each strain. Expression of any of the H. influenzae or *Neisseria nfsB* homologs was sufficient to significantly increase the resistance of Es. coli to chloramphenicol (*q* < 0.05 to *q* < 0.0005), while expression of the P. multocida *nfsB* resulted in a negligible but significant increase in susceptibility instead (*q* < 0.0005) (Fig. 1e). The relative increase in resistance conferred by H. influenzae or *Neisseria nfsB* expression ranged from approximately a 16-fold increase (Hi KW20 and Hi 86-028NP) down to an ∼2-fold increase (Hi Int1 and Hi 12). The chloramphenicol MICs for each strain followed this same pattern (Fig. S2C).

### Liquid chromatography mass spectrometry analysis of NfsB chloramphenicol reduction products.

We next overexpressed and purified NfsB enzymes for *in vitro* study. Each *nfsB* homolog was cloned into a new inducible plasmid for overexpression in Es. coli. Each histidine-tagged construct was purified by affinity chromatography followed by enzymatic removal of histidine tags. All four H. influenzae NfsB enzymes and Nm Z2491 NfsB expressed well as soluble proteins, while the Ng FA1090 and Pm 70 enzymes resulted in production of insoluble protein.

**FIG 2:**
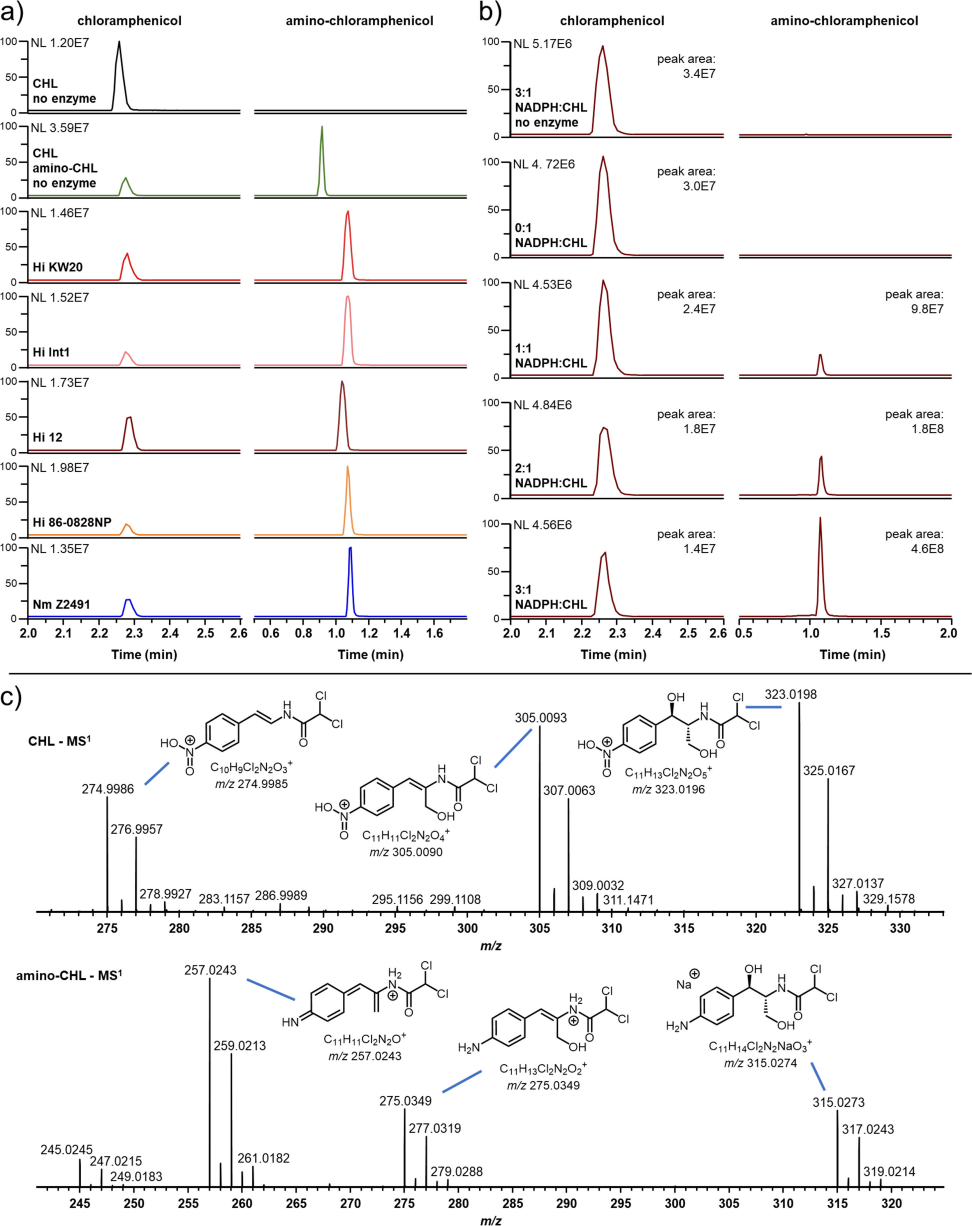
*In vitro* chloramphenicol reduction products. Extracted ion chromatograms showing *in vitro* reduction of chloramphenicol (CHL) to amino-chloramphenicol (amino-CHL) by five NfsB homologs (a) and reduction of chloramphenicol to amino-chloramphenicol by NfsB Hi 12 with increasing molar equivalents of NADPH compared to chloramphenicol (b). Reactions in panels a and b were monitored by measuring peak areas of the chloramphenicol [M + H]^+^ ion at *m/z* 323.0196 and the amino-chloramphenicol [M + Na]^+^ ion at *m/z* 315.0274. (c) MS^1^ spectra for chloramphenicol (top) and amino-chloramphenicol (bottom) with the predicted structures of their gas-phase dehydration product ions (see Fig. S3 in the supplemental material for alignment of the MS^1^ ions’ extracted ion chromatograms).

We used liquid chromatography mass spectrometry (LCMS) to determine if the H. influenzae and N. meningitidis NfsB enzymes reduce chloramphenicol to amino-chloramphenicol. The absence of standards for unstable reduction intermediates, such as nitroso- and hydroxylamino-chloramphenicol, prevented the targeted detection of these compounds. We set up minimal enzymatic reactions (only enzyme, chloramphenicol, and NADPH) with excess equivalents of NADPH (previously shown to be the preferred electron donor of Hi KW20 NfsB [15]) and analyzed them by LCMS for the formation of amino-chloramphenicol. While excess flavin mononucleotide (FMN) was added to other reactions to ensure near complete cofactor occupancy, it was also omitted here to avoid potential matrix effects. All five enzymatic reactions resulted in robust amino-chloramphenicol peaks, while this product was not detectable in the “no enzyme” control (Fig. 2a). Despite the presence of excess NADPH (four equivalents versus the three theoretically necessary) (Fig. 1a), the chloramphenicol substrate was not entirely depleted in these reactions. We therefore performed a series of reactions with stepwise increases in NADPH equivalents using the enzyme Hi 12 NfsB. Each increase in NADPH equivalents led to qualitatively larger amino-chloramphenicol and smaller chloramphenicol peaks (Fig. 2b).

Following optimization of instrument parameters, the molecular ions for chloramphenicol and amino-chloramphenicol were detected at an [M + H]^+^ of *m/z* 323.0198 and an [M + Na]^+^ of *m/z* 315.0274, respectively (Fig. 2c). In addition, we detected prevalent ions that represent gas-phase reaction products for both amphenicol species in the MS^1^ spectra. Electrospray ionization of chloramphenicol yielded [M + H]^+^ ions for its derivative allylic alcohol at *m/z* 305.0090 and its derivative nitrostyrene at *m/z* 274.9985. Ionization of amino-chloramphenicol yielded [M + H]^+^ ions for its derivative allylic alcohol at *m/z* 275.0349 and its derivative iminoquinone methide at *m/z* 257.0243 (Fig. 2c; Fig. S3). We did not have *N*-hydroxylamino-chloramphenicol or nitroso-chloramphenicol standards for comparison, and we did not observe ions that corresponded to the expected masses of these reactive intermediates.

### NfsB chloramphenicol reduction and NADPH oxidation kinetics.

**FIG 3:**
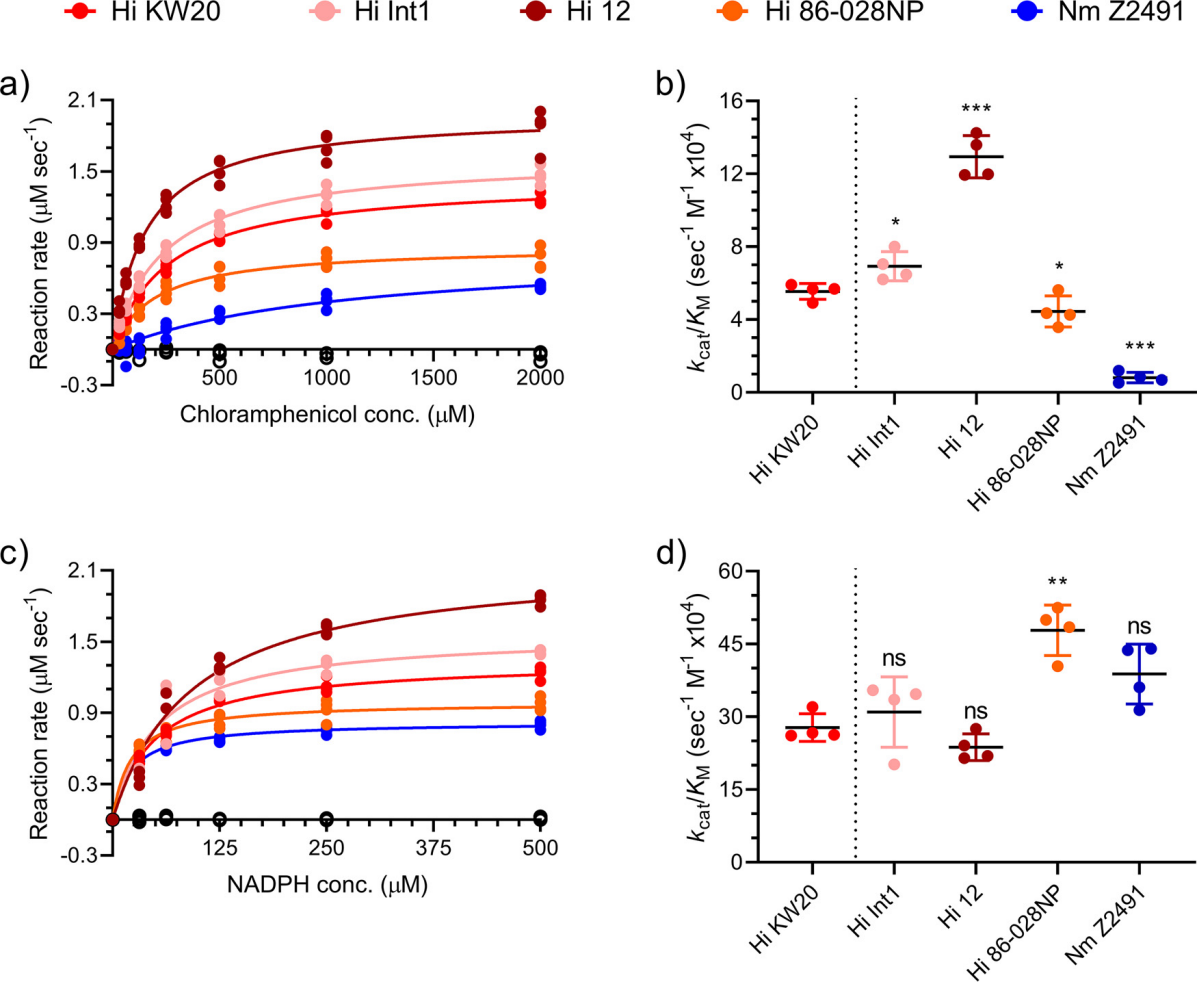
Michaelis-Menten kinetics of NfsB chloramphenicol reduction and NADPH oxidation. (a) Quadruplicate (*n* = 4) Michaelis-Menten chloramphenicol reduction curve fits were used to estimate *k*_cat_ and *K_m_* values with standard deviation (Table 1) for each NfsB enzyme; conc., concentration. (b) Chloramphenicol reduction kinetic efficiency (*k*_cat_/*K_m_*) values were calculated as means with standard deviation error bars (*n* = 4) of independent curve fit analyses. Significance was determined in comparison to the Hi KW20 enzyme by Brown-Forsythe and Welch ANOVA testing with Benjamini, Krieger, and Yekutieli false-discovery rate correction (ns, not significant; *, *q* < 0.05; **, *q* < 0.005; ***, *q* < 0.0005). (c) Quadruplicate (*n* = 4) Michaelis-Menten NADPH oxidation curve fits were prepared. (d) NADPH oxidation kinetic efficiency (*k*_cat_/*K_m_*) values were calculated as in panels a and b above.

In the process of standardizing conditions for reactions to be monitored by LCMS, we noticed that *in vitro* chloramphenicol reduction progress curves suggested that the Hi 12 and Hi Int1 NfsB enzymes had greater activity than the other enzymes, including Hi KW20 NfsB (Fig. S4). This contrasts with our Es. coli-based assays (Fig. 1c and e; Fig. S2A). We therefore measured chloramphenicol reduction kinetics and found that all five enzymes showed initial velocity versus substrate concentration relationships consistent with Michaelis-Menten kinetics (Fig. 3a). We compared the activity of each NfsB homolog against Hi KW20 NfsB because it was the only previously characterized chloramphenicol reductase. Hi 12 NfsB showed both the fastest apparent maximum rate and, overall, significantly higher activity (>2-fold more active; *q* < 0.005) than Hi KW20 NfsB as measured by catalytic efficiency (*k*_cat_/*K_m_*). The Hi Int1 and Hi 86-028NP enzymes showed comparable, although statistically significant, kinetics (*q* < 0.05) to Hi KW20 NfsB. In contrast, Nm Z2491 NfsB was much less active *in vitro*, with ∼15% the activity as Hi KW20 NfsB (*q* < 0.0005), apparently driven by a much larger *K_m_* value (Fig. 3b and Table 1).

**TABLE 1:** Michaelis-Menten kinetics of chloramphenicol reduction and NADPH oxidation

NfsB homolog	Substrate	*k*_cat_ (s^−1^)^*a*^	*K_m_* (μM)^*a*^	*k*_cat_/*K_m_* (M^−1^ s^−1^)
Hi KW20	Chloramphenicol	14 ± 0.3	260 ± 20	5.55 × 10^4^
	NADPH	13 ± 0.5	49 ± 6	2.78 × 10^5^
Hi Int1	Chloramphenicol	16 ± 1	240 ± 30	6.93 × 10^4^
	NADPH	16 ± 0.7	54 ± 20	3.10 × 10^5^
Hi 12	Chloramphenicol	20 ± 2	160 ± 20	1.29 × 10^5^
	NADPH	22 ± 0.7	93 ± 10	2.37 × 10^5^
Hi 86-028NP	Chloramphenicol	8.7 ± 0.9	200 ± 40	4.45 × 10^4^
	NADPH	9.9 ± 0.6	21 ± 2	4.78 × 10^5^
Nm Z2491	Chloramphenicol	8.9 ± 3	1,300 ± 800	8.12 × 10^3^
	NADPH	8.2 ± 0.3	22 ± 4	3.88 × 10^5^

a Values reflect mean ± standard deviation values from four independent reactions.

We next measured the kinetics of the NADPH oxidation reaction (Fig. 3c). In contrast to the reduction of chloramphenicol, the oxidation of NADPH showed less apparent variability in catalytic efficiency, with only Hi 86-028NP NfsB showing significantly greater activity than Hi KW20 NfsB (∼1.7-fold, *q* < 0.005) (Fig. 3d and Table 1). As expected, the apparent *k*_cat_ values for NADPH oxidation closely match those for chloramphenicol reductions, while the *K_m_* values differed significantly (Table 1).

### Structural underpinnings of Hi 12 NfsB increased chloramphenicol reduction activity.

**FIG 4:**
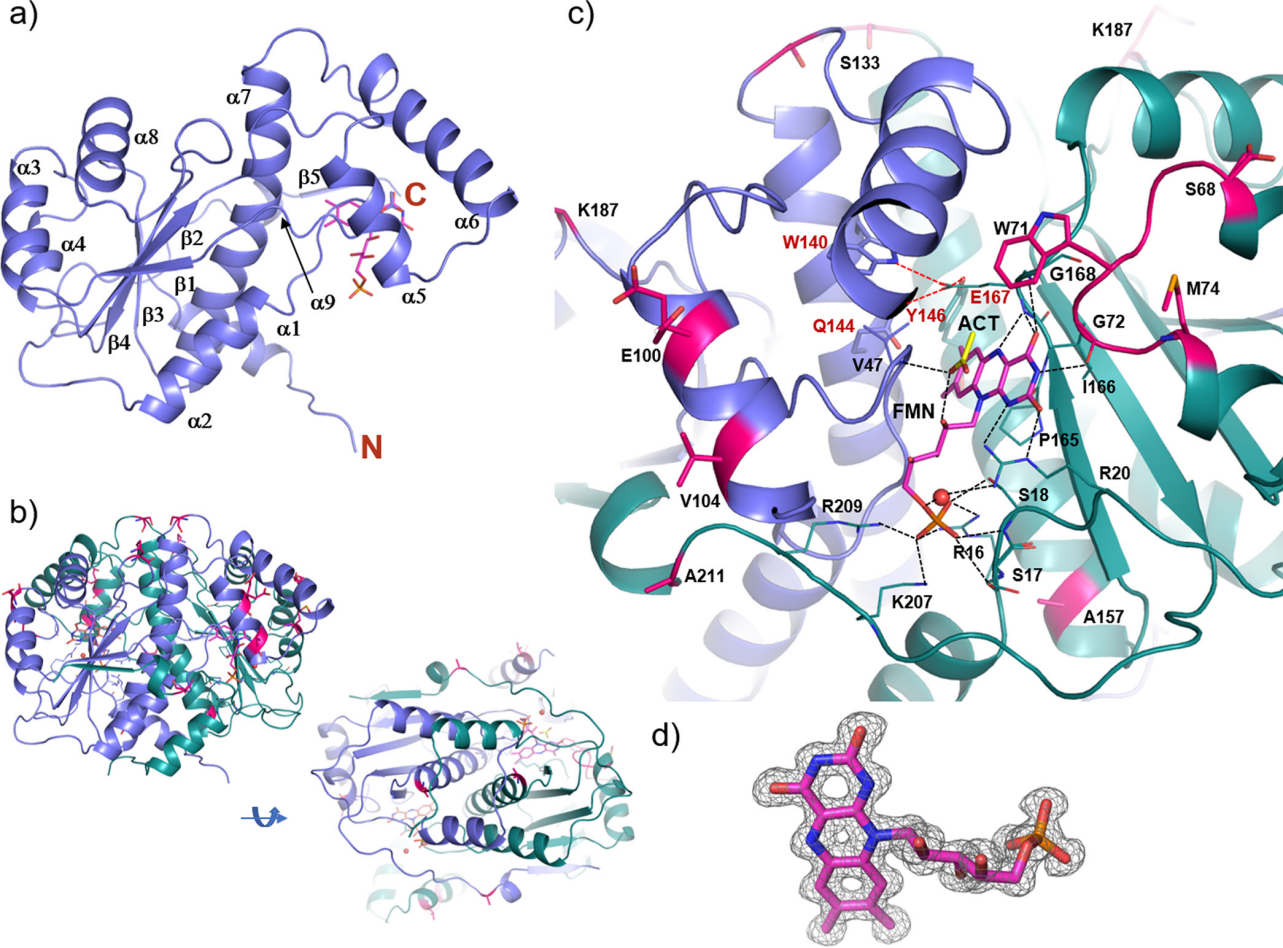
Crystal structure of Hi 12 NfsB in complex with an FMN prosthetic group. Monomeric (a) and dimeric (b) Hi 12 NfsB X-ray crystal structures in two different views 90° apart. (c) Close-up view of the dimer interface/substrate-binding site. The flavin mononucleotide (FMN) prosthetic group is shown as a magenta stick model, and a bound acetate (ACT) molecule is shown as a yellow stick model. Residues interacting with FMN are shown as thin sticks, and the corresponding hydrogen bonds are indicated by black dashed lines. Red dashed lines indicate hydrogen bond interactions between E176 and Y146 from the teal monomer and W140 and Q144 from the purple monomer. Residues shown with red backbones represent residues not conserved in Hi 12 NfsB compared to other H. influenzae NfsB homologs (see the text and Fig. S5 in the supplemental material). (d) Well-defined electron density (2*F*_o_ – *F*_c_, 1.5*σ*) of the FMN prosthetic group.

We next solved the structure of Hi 12 NfsB, the most active enzyme *in vitro*. The 1.15-Å structure (Fig. 4; Table S3) shows that the protein belongs to the NADH oxidase superfamily (CATH 3.40.109.10), making it the highest resolution experimental model of this superfamily. These proteins belong to α/β-class with 3-layer sandwich architecture. The 220-residue protein folds into a very well-ordered α/β/α-sandwich that consists of nine α-helices and five β-strands (Fig. 4a and b). Two monomers oligomerize and form a classic symmetric nitroreductase dimer. In each protomer, the five-stranded twisted β-sheet (with the fifth strand [β5] coming from the C-terminal part of the symmetry-related protomer) is surrounded by six helices, three (α3, α4, and α8) from the outer side of the dimer and three (α2, α7, and α9) from the other side. The interacting surfaces provided by protomers are complementary in terms of shape and charge. The longest helix α7 (residues 135 to 160), α2 (residues 30 to 42), and the loop leading to β1 from both protomers symmetrically form the extensive dimer interface (10,410 Å^2^ solvent-excluded surface), which also includes the binding pocket for a substrate (Fig. 4b and c) and the well-resolved FMN prosthetic group (Fig. 4d). The C-terminal residues 211 to 220, including the three-quarter turn helix α9 and the short β5 strand, go over and tightly wrap around the other protomer, with β5 forming the complete five-stranded β-sheet with four β-strands from the other protomer. The binding pocket, located next to β3, is composed of the loop between α1 and α2 (residues 16 to 20), the C-terminal part of β3 and the loop leading to α8 (residues 165 to 169), the short turn between α3 and α4 from the protomer 1 (residues 71 to 74), and the loop between α2 and β1 from the second protomer (residues 43 to 47). In the binding site, the isoalloxazine ring of FMN interacts through hydrogen bonds with the carbonyl oxygen of G72 through N3 (2.8 Å), with NH2 of R20 through N1 (3.1 Å), with N of E167 through N5 (2.9 Å), and with NE of R20 (2.7 Å), with N of G168 (2.9 Å) and N of E167 through O4 (3.4 Å) (Fig. 4c). All the protein residues mentioned here are strictly conserved (Fig. S5). O2′ and O5′ of the FMN ribityl tail interact with R20 NH2 (3.0 Å) and, through a water-mediated hydrogen bond, NH1.

The FMN phosphoryl oxygens are also involved in hydrogen-bonding interactions with strictly conserved protein residues: O2P with NH1 (3.0 Å) and NH2 (3.1 Å) of R16 and with OG of S18 (2.7 Å); O3P with N of S18 (2.8 Å) and with OG of S17 (3.2 Å); and O1P with NH2 of R16 (3.2 Å), with NZ of K207 (2.8 Å), and with NH2 of R209 (2.7 Å). There are additional van der Waals contacts through P165 and from the second protomer with P44. The acetate molecule, a small substrate analog, is on the “*re”*-side (over flavin) of the isoalloxazine ring. It makes hydrogen bonds with O2′ of the FMN ribityl tail (2.7 Å) and N (2.8 Å) of V47 from the second monomer (Fig. 4c). The protein residues involved in interactions with FMN are mostly from one protomer and are on the “*re”*-side of FMN.

**FIG 5:**
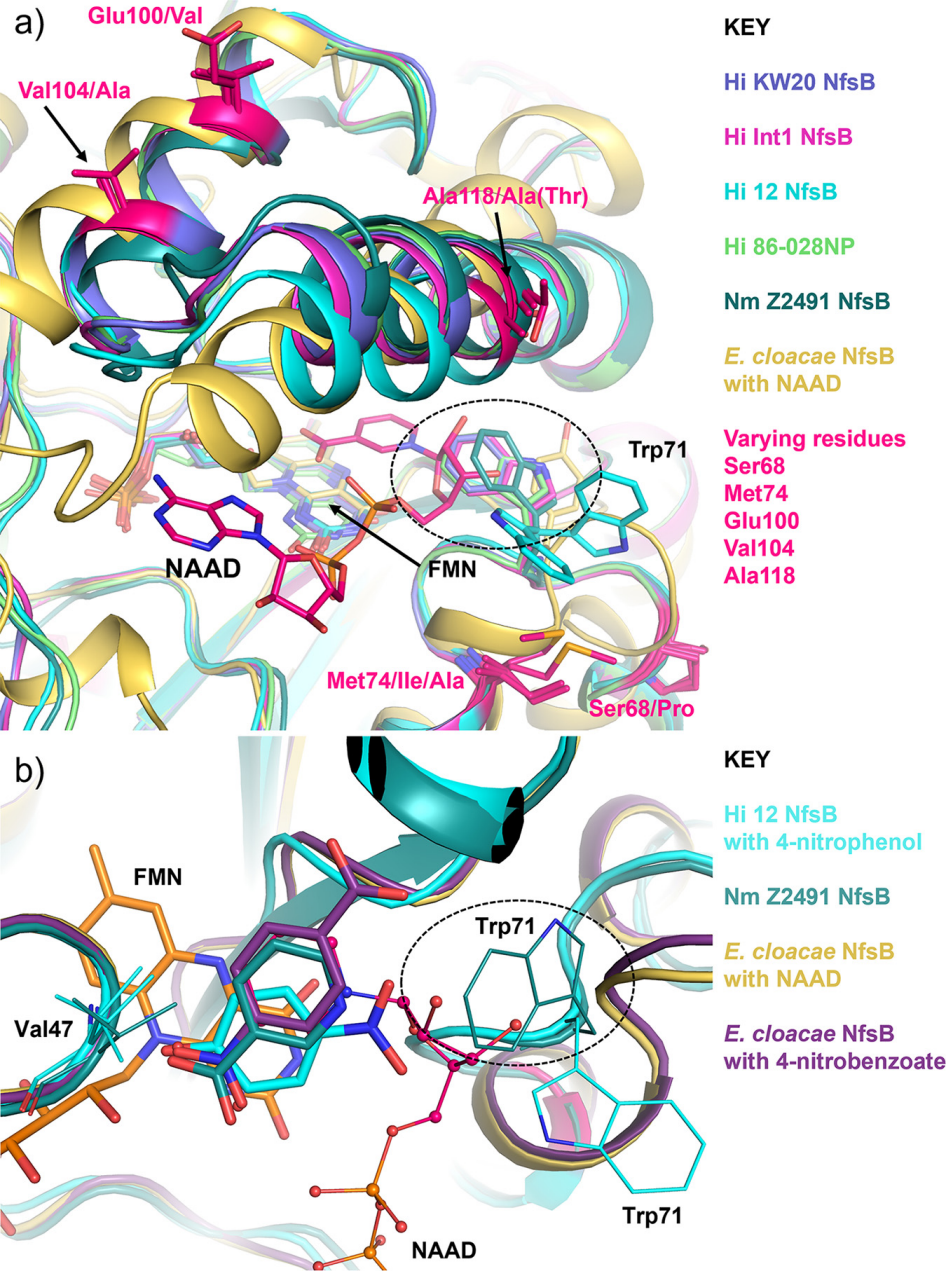
Structural comparison of NfsB active sites with relevant substrates. (a) Probable NADP binding in NfsB active site. Hi KW20 NfsB is in purple, Hi Int1 NfsB is in magenta, Hi 12 NfsB is in cyan, Hi 86-028NP NfsB is in light green, Nm Z2491 NfsB is in dark green, and En. cloacae NfsB in the complex with NAAD (elements: N, blue; O, red; C, hot pink) is in gold. Respective PDB IDs are 7S1A, 7RZP, 7RZL, 7S14, 6WT2, and 5J8D (21). Residues showing variation among H. influenzae and Neisseria meningitidis NfsB homologs are shown in hot pink. The potential clash between the ribose of NADP^+^ and Nm Z2491 NfsB Trp71 is indicated with the dashed circle. (b) Ligand binding in the NfsB active site. Hi 12 NfsB complexed with 4-nitrophenol is in cyan, Nm Z2491 NfsB is in dark green, En. cloacae NfsB complexed with NAAD is in gold, and En. cloacae NfsB complexed with 4-nitrobenzoate is in deep purple. Respective PDB IDs are 7RZL, 6WT2, 5J8D, and 5J8G (21). The Trp71 side chain from Hi 12 is flipped to avoid clashing with 4-nitrophenol. The potential clash between the ribose of NADP^+^ and Trp71 of Nm Z2491 NfsB is shown as a dashed oval.

To understand better how variation in the substrate-binding site mediates activity, we solved the crystal structures of the NfsB homologs from Hi KW20, Hi Int1, Hi 86-028NP, and Nm Z2491 (Fig. 5a). We also soaked an analog of chloramphenicol, 4-nitrophenol, into Hi 12 NfsB crystals and solved the resulting enzyme-substrate complex (Fig. 5a and b).

Including the 4-nitrophenol-bound form of Hi 12 NfsB, the root mean square deviation (RMSD) of Cα atoms among these structures are very small with a range of 0.36 to 0.61 Å for the H. influenzae proteins and 0.9 Å for the Nm Z2491 structure. To test the hypothesis that the observed catalytic differences (Fig. 3) arise from structural adjustments during NADP/NADPH cycling, we compared these structures with previously solved nitroreductases from Enterobacter cloacae complexed with nicotinic acid adenine dinucleotide (NAAD, an analog of NADP) (PDB: 5J8D) or 4-nitrobenzoic acid (comparable to chloramphenicol and 4-nitrophenol) (PDB: 5J8G) (21). The main structural differences in Hi 12 NfsB regarding NADP binding are the short loop between α3- and α4-containing residues Ser68-Met74 (including Trp71) and the segment between α5- and α6-containing residues 99 to 125 (Fig. 5a). These regions correspond to the few amino acid positions that differ between Hi 12 NfsB and the other Hi homologs (Fig. S5). The Hi 12 NfsB unconserved residues E100(V), V104(V), and A118(A/T) are all in the middle of the helices themselves and do not appear to play an important role in structure adjustment. Conversely, the ribose of NADP must overlap the Trp71 side chain, and the loop between α5 and α6 needs to collapse down to the adenine moiety of NADP (or NAAD in the En. cloacae structure [5J8D]). Similarly, when Hi 12 NfsB contains 4-nitrophenol in its active site, Trp71 swings out, with movement potentially of the whole α3-to-α4 loop, to avoid a clash with the 4-nitrophenol NO_2_ group. This movement appears to be supported through an interaction with the Met74 side chain (Fig. 5a), in which the S (Sδ) of Met attracts the Trp71 aromatic ring through a long π-π interaction (7 to 10 Å) (22).

For structural changes specific to substrate binding (e.g., nitroaromatics), as opposed to coenzyme binding (e.g., NADPH), we focused on comparisons between the Hi 12 NfsB structure complexed with 4-nitrophenol, the Nm Z2491 structure complexed with nicotinic acid, and the previously published En. cloacae structures complexed with 4-nitrobenzoic acid or NAAD (Fig. 5b). The phenyl rings of nicotinic acid in the Nm Z2491 NfsB structure and 4-nitrobenzoic acid in the En. cloacae structure coincide with the niacin ring of NAAD in the other En. cloacae structure. The 4-nitrophenol molecule, complexed with Hi 12 NfsB, takes the same position, although rotated approximately 50° around its hydroxy oxygen. This places the NO_2_ group in the opposite orientation of the 4-nitrobenzoic acid NO_2_ group complexed with the En. cloacae enzyme, although the orientation of the NO_2_ and the CO_2_ groups of the 4-nitrobenzoic acid molecule were not clearly defined (21). These three ligands (4-nitrophenol, nicotinic acid, and 4-nitrobenzoic acid) each assume the position and orientation of the niacin ring of NAAD (Fig. 5b). This suggests that substrate and the niacin of NADP bind the same location relative to FMN in the enzyme-binding site at the different catalytic time points.

## DISCUSSION

Antimicrobial resistance is a present and growing concern (2), and novel mechanisms of antibiotic resistance are especially alarming (4). One potential source of new antimicrobial resistance enzymes is housekeeping enzymes with promiscuous substrate use. If their substrate range includes antimicrobials, they are considered cryptic or proto-resistance enzymes (i.e., a protein without the ability to confer phenotypic resistance in its original context but having the potential to confer resistance through mutation and/or changes in expression [23]). This may be the case even if substrate turnover and specificity remain low or the corresponding gene remains transcriptionally independent of antimicrobial exposure (4, 23). We previously demonstrated that expression of the *nfsB* housekeeping gene from H. influenzae KW20 (Hi KW20) is sufficient to confer clinically relevant levels of chloramphenicol resistance in Es. coli (15). This satisfies the definitions of cryptic and proto-resistance genes and shows potential for clinical relevance through mobilization/change in context and change in expression (23). Here, we built upon this initial observation (15–17) to survey the functional landscape of potential chloramphenicol reductases across a range of amino acid identities.

Expression of *nfsB* homologs from other H. influenzae strains as well as *Neisseria* species in Es. coli conferred different levels of chloramphenicol resistance (Fig. 1e), with the highest level of resistance coming from expression of H. influenzae KW20 *nfsB*. We established the role of nitroreduction as critical by showing that resistance was specific to nitro group-containing amphenicols (Fig. 1d). The lack of significant chloramphenicol resistance or reduction in our empty vector control strain (Fig. 1c to e) suggests that the native Es. coli *nfsB* does not use chloramphenicol as a substrate. This is consistent with our previous findings that overexpression of the Es. coli *nfsB* does not confer increased chloramphenicol resistance in Es. coli (15) and suggests that use of chloramphenicol as a substrate is not a universal feature of NfsB enzymes.

All the enzymes tested converted chloramphenicol to amino-chloramphenicol *in vitro* (Fig. 2a), and we discovered that the NfsB homolog from Hi 12 is significantly more active than the previously studied Hi KW20 homolog *in vitro* (Fig. 3b and Table 1). The divergence in chloramphenicol resistance and *in vitro* activity likely reflect the vagaries of heterologous expression in Es. coli and suggest the presence of important subtleties that still need to be accounted for when estimating mobilization risks of cryptic and proto-resistance genes. This is especially evident with the NfsB enzymes from Hi KW20 and Hi Int1. These homologs share 99.09% amino acid identity (Table S1 in the supplemental material) and similar *in vitro* chloramphenicol reduction kinetics (Table 1) but confer very different levels of chloramphenicol resistance when expressed in Es. coli (Fig. 1e). A molecular explanation for this difference remains to be determined. However, while the H. influenzae 12 NfsB retains greater than 94.98% identity with the other H. influenzae NfsB homologs (Table S1), it showed a significantly greater catalytic efficiency *in vitro* (Fig. 3b and Table 1) despite its ability to confer resistance in Es. coli being less dramatic (Fig. 1e; Fig. S2C) and likely tempered by other factors relevant to heterologous expression.

In the initial identification of amino-chloramphenicol as a product of H. influenzae cultures incubated with chloramphenicol (12), Smith et al. proposed that it was just one component in a set of interconverting species. We observed the same set of amino-chloramphenicol-related species in our mass spectrometry experiments (Fig. 2c). However, our data suggest that the additional species are experimental artifacts, specifically gas-phase dehydration reaction products that arise from positive-mode LCMS electrospray ionization rather than the enzymatic reaction. Our reasoning is 3-fold. First, we observed amino-chloramphenicol dehydration product ions in all LCMS runs, including with a pure amino-chloramphenicol standard (Fig. 2c). This suggests that they are abiotic. Second, the ions in question arise from the same chromatographic peak as their parent molecules. Given the likely differences in polarity of these compounds, it would be highly unlikely for them to share the same chromatography retention times (Fig. S3). Third, the supplier of our amino-chloramphenicol standard (Toronto Research Chemicals) provided positive-mode LCMS data containing the same dehydration product ions, while their negative-mode LCMS data yielded only the free base and chloride adduct, and their ^1^H-nuclear magnetic resonance (^1^H-NMR) spectrum revealed pure amino-chloramphenicol (data not shown). We are therefore confident that abiotic gas-phase dehydration reactions during LCMS analysis are responsible for the formation of the amino-chloramphenicol-related metabolites and not additional enzymes in an uncharacterized pathway or nonenzymatic processes as originally hypothesized (12).

Nitroreductase enzymes have been found to be able to turnover a variety of substrates (24). In characterizing the *in vitro* reduction of chloramphenicol, we wanted to measure to what degree this activity reflects promiscuity in NfsB enzymes as opposed to other traditional antibiotic resistance enzymes. Historically, the NfsB enzyme family was named for their phenotypic connection to nitrofuran sensitivity (25), and Es. coli NfsB has been found to use nitrofurans as substrates with *K_m_* values that suggest a reasonable degree of substrate specificity. For example, Zenno et al. recorded a *K_m_* of 153 µM for nitrofurazone (26), which is close to the estimated median bacterial enzyme *K_m_* of 130 µM (27). Linwu et al. similarly found that Es. coli NfsB has *K_m_* values for nitrobenzodiazepines ranging from ∼6 µM to ∼40 µM. We found that the NfsB homologs studied here have *K_m_* values for chloramphenicol ranging from 160 µM for Hi 12 NfsB to 1,300 µM for Nm Z2491 NfsB (Table 1), although all enzymes showed apparent *k*_cat_ values near the median enzyme value of 13.7 s^−1^ (∼9 s^−1^ to ∼22 s^−1^; Table 1). In our previous work, we measured *K_m_* values for Hi KW20 NfsB with several substrates and found them to range from a low of ∼40 µM for nitrofurantoin to a high of ≥750 µM for 4-nitrophenol (15). In their thorough analysis of the Hi KW20 NfsB substrate range, Green et al. found significant nitroreduction activity with some but not all nitroaromatic substrates, with chloramphenicol, 4-nitrophenol esters, 4-nitrophenol ethers, and substituted nitronaphthalenes showing the greatest turnover (17). Together, these observations support the hypothesis that chloramphenicol reduction may represent an emergent activity within a broadly promiscuous enzyme class.

The apparently moderate *in vitro* activity of these enzymes is still sufficient to confer significant levels of resistance when expressed in Es. coli. For example, the N. meningitidis NfsB enzyme has a comparatively low *k*_cat_/*K_m_* of ∼8 × 10^3^ M^−1 ^s^−1^ for chloramphenicol *in vitro* (Table 1). When expressed in Es. coli, however, it confers an ∼8-fold increase in IC_50_ and an MIC double the clinical resistance cutoff of 8 µg/mL (Fig. 1e; Fig. S2C). While the chloramphenicol reduction *k*_cat_/*K_m_* values measured here (Table 1) are lower than diffusion-limited resistance enzymes (e.g., β-lactamases, ∼10^8^ M^−1 ^s^−1^), they are in line with other reported canonical antibiotic-modifying enzymes, including aminoglycoside acetyltransferases and phosphotransferases (e.g., ∼2 × 10^3^ M^−1 ^s^−1^ to ∼3 × 10^4^ M^−1 ^s^−1^) (27, 28), suggesting that less kinetically “perfect” antimicrobial-modifying enzymes are sufficient to overcome bacteriostatic antibiotics. Similarly, while a natural substrate for NfsB is not known, some *nfsB* homologs are regulated by the MarA transcriptional activator, part of the multiple antibiotic resistance (*mar*) locus. This suggests that in some contexts, *nfsB* homologs play a role in bacterial responses to antibiotics, pollutants, and other xenobiotics (24).

Previous kinetic studies of nitroreductase catalysis revealed that reactions progress by a double-displacement (ping-pong) mechanism that appears to lack the gating steps necessary to impose specificity, explaining the substrate promiscuity of these enzymes. In this mechanism, two electrons are transferred as a hydride from NAD(P)H to the oxidized FMN and from reduced anionic FMN to substrate (Fig. 1a) (21). Hydride transfer can occur when specific geometry and distances are preserved, with the optimal distance between the donor and recipient atoms being ∼3.8 Å. In our three-dimensional crystal structure of Hi 12 NfsB (Fig. 4), an acetate molecule binds in the same orientation as potential substrates at a distance of 3.6 Å, making it compatible with the hydride transfer hypothesis.

In exploring the structure-function foundations of chloramphenicol reduction by NfsB enzymes, we noted that Hi 12 NfsB differs from each of the other H. influenzae NfsB homologs at only eight amino acid positions (Fig. S5). Among these, residues S68 and M74 appear to be particularly well positioned to influence substrate binding (Fig. 4). To explore this further, we solved the crystal structures of several other chloramphenicol-reducing NfsB enzymes (Fig. 5). With amino acid sequence identities of greater than 94.6% between NfsB proteins from H. influenzae strains (Table S1), the *in vitro* activity differences of these proteins (Fig. 3 and Table 1) are somewhat surprising. Because the NADPH and nitro substrate-binding sites overlap (Fig. 4 and 5), the binding of NADPH and a nitro substrate to individual subunits must occur sequentially, with NADPH binding first to reduce FMN to FMNH_2_ by hydride transfer before being released as NADP^+^. This is followed by binding of the nitro substrate, which can then be reduced by FMNH_2_ (Fig. 1a). These overlapping binding sites and stepwise reaction progression suggest that differences in chloramphenicol reduction may come from NADPH processing steps where enzyme structural adjustments at or near the binding site are necessary for catalysis. Previous structural studies of other NfsB enzymes confirm that residues between α3 and α4 (homologous to Ser68 to Met74 in Hi 12 NfsB) are positioned to influence substrate binding (29, 30). Our crystal structures of Hi 12 NfsB with and without a nitroaromatic substrate (Fig. 4 and 5) and others’ previously published En. cloacae NfsB structures support this hypothesis by demonstrating the overlap of Trp71 with this binding pocket and its potential to sterically clash with substrates (Fig. 5). Intuitively, the Ser68-Met74 pair found in Hi 12 NfsB would provide more of the flexibility needed to properly locate and orient Trp71 to make space for the ribose of NADP than the Pro68-Ile74 pair in other H. influenzae NfsB homologs (Pro68-Ala74 in Nm Z2491 NfsB). A glycine residue at the equivalent position in En. cloacae NfsB has similarly been hypothesized to provide greater loop flexibility (21).

**FIG 6:**
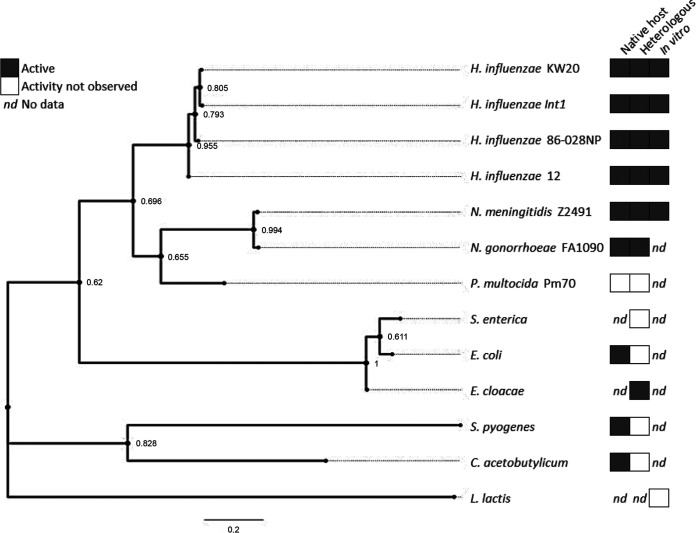
Summary of NfsB chloramphenicol reduction activity across phyla. Maximum likelihood tree of NfsB homologs from the indicated taxa with L. lactis CinD (NfsA homolog) as the outgroup. Chloramphenicol reduction activity in the originating organism (“native host”), in Es. coli expressing the gene (“heterologous”), or in an enzymatic assay (“*in vitro*”) is indicated to the right, with dark boxes indicating significant activity and white boxes indicating no activity detected (see Table S4 in the supplemental material for corresponding citations 9, 11–12, 15–17, 56); nd, corresponds to data that are not available.

To our knowledge, just over a dozen NfsB/NfsA enzyme homologs, including those from H. influenzae, N. meningitidis and N. gonorrhoeae, P. multocida, Salmonella enterica, Es. coli, Enterobacter cloacae, Streptococcus pyogenes, Clostridium acetobutylicum, and Lactococcus lactis, have been studied for their ability to turn over chloramphenicol as a substrate. We have attempted to summarize the current state of this research in Fig. 6. These studies (Table S4) have included assays for chloramphenicol reduction in pure culture (native host), in Es. coli cultures with *nfsB* heterologous expression, and *in vitro* with purified enzymes. To the best of our knowledge, outside the manuscript and three publications that mostly focused on the Hi KW20 NfsB (15–17), this class of genes and enzymes have not been extensively assayed for chloramphenicol modification. This makes it difficult to extrapolate how widespread this activity may be. We found that expression of the P. multocida *nfsB* did not result in amino-chloramphenicol production or confer chloramphenicol resistance in E. coli (Fig. 1c and e; Fig. 2a and c), despite the corresponding enzyme clustering with active Haemophilus and *Neisseria* homologs. In contrast, in our previous work, we found that expression of En. cloacae *nfsB* did confer significantly increased chloramphenicol resistance in Es. coli, despite the lack of resistance conferred by the closely related Sa. enterica and Es. coli genes (15). These two pieces of evidence suggest that chloramphenicol reductase activity in NfsB enzymes may not be predictable through taxonomy or sequence similarity alone, although we propose that ample additional examples of NfsB homologs with chloramphenicol reductase activity remain to be characterized outside these taxa. We suggest that the study of these homologs would make an attractive model for the steps required to evolve housekeeping enzymes into resistance enzymes. Recently, a mutated Es. coli NfsA enzyme was reported to show chloramphenicol reductase activity (18). Its ∼150-fold lower catalytic efficiency than Hi 12 NfsB for this reaction and the total lack of chloramphenicol reductase activity reported by Mermod et al. for another NfsA homolog (L. lactis CinD) (31) suggest that comparisons of NfsA to NfsB enzyme families may similarly yield novel insights.

In summary, we have demonstrated that NfsB nitroreduction of chloramphenicol to amino-chloramphenicol is not limited to the single published example of H. influenzae strain KW20 NfsB but is distributed across multiple taxa (Fig. 6). These new *nfsB* homologs confer increased resistance to chloramphenicol when expressed in Es. coli and show *in vitro* chloramphenicol reduction kinetics consistent with some antibiotic resistance enzymes. We have identified that the NfsB homolog from H. influenzae strain 12 exhibits the highest chloramphenicol reduction activity thus far, likely due to increased flexibility in the small loop between α3 and α4 in the active site. We propose that additional NfsB homologs be studied as potential models for housekeeping enzymes that may be evolvable into resistance enzymes.

## MATERIALS AND METHODS

### Materials.

Chloramphenicol (≥98%; C0378-5G), thiamphenicol (>99.9%; T0261-15), and kanamycin sulfate (KAN) (K1377) were purchased from Sigma-Aldrich and prepared as 50 mg/mL stock solutions in ethanol (amphenicols) or water, filter sterilized, and stored at −20°C. Bratton-Marshall reagent (*N*-[1-naphthyl]ethylenediamine HCl) (98+%; AC423990250), sodium nitrite (97% minimum; AA1424422), ammonium sulfamate (98+%; AC423390050), riboflavin 5′-monophosphate sodium salt (FMN) (93.0+%; R00235G), and florfenicol (NC1617017) were purchased from Fisher Scientific and prepared as described below. β-Nicotinamide reduced tetrasodium salt (NADPH) was purchased from Dot Scientific (>93%; DSN20140-0.1), stored dry at −20°C, and prepared fresh for all experiments. Amino-chloramphenicol was purchased from Toronto Research Chemicals (A622670) as an HCl salt and prepared by dissolution in water. Amino-chloramphenicol solutions were stored at −20°C for less than 1 month. Sch-24893 (Fig. 1b) was synthesized by the Northwestern University ChemCore, validated by proton and carbon NMR in dimethyl sulfoxide (DMSO) (Fig. S1A and B), and prepared as a 50 mg/mL stock solution in ethanol.

### General culture conditions.

Routine cultivation of Escherichia coli was performed aerobically at 37°C in LB medium or on LB agar plates that contained 10 g of tryptone, 5 g of yeast extract, and 10 g of NaCl per liter. When cultured for antibiotic resistance testing, Es. coli strains were grown aerobically at 37°C in Mueller-Hinton cation-adjusted medium (MH medium) (BD BBL, 212322). When necessary for plasmid maintenance, the antibiotic kanamycin (KAN) was included at 50 µg/mL final concentration (KAN50; i.e., LB + KAN50 or MH + KAN50).

### Analysis of NfsB sequences and maximum likelihood tree.

Homologs of the H. influenzae Rd KW20 (Hi KW20) NfsB enzyme were identified in the genomes of H. influenzae strains Hi Int1, Hi 12, and Hi 86-028NP and from the genomes of Neisseria gonorrhoeae FA1090, Neisseria meningitidis Z2491, and Pasteurella multocida Pm70 by reciprocal protein BLAST search (32). Protein BLAST alignments were also used to determine pairwise percent amino acid identity between each homolog (default settings).

Amino acid sequences were downloaded for the above NfsB homologs, previously studied homologs (15) from Streptococcus pyogenes, Clostridium acetobutylicum, Salmonella enterica, Enterobacter cloacae, and Es. coli as well as the more distantly related NfsA homolog CinD from Lactococcus lactis (31). All 13 sequences were aligned by MUSCLE (33) in the MegaX program (34–36) with default parameters: gap open −2.9, gap extend 0, H-phobic multiplier 1.2, cluster method iterations 1,2 UPGMA, other iterations UPGMA, min diag length (lambda) 24. The appropriate maximum likelihood model for this data set was determined by MegaX to be the LG+G model. A maximum likelihood tree was built using this model with 1,000 bootstrap support and L. lactis CinD assigned as outgroup.

### Cloning *nfsB* genes for phenotypic study in Es. coli.

Open reading frames (ORFs) of the genes selected for study were codon optimized for expression in Es. coli and synthesized by Integrated DNA Technologies. The following NAD(P)H-flavin oxidoreductases with corresponding GenInfo Identifier (gi) number were targeted for study: H. influenzae Rd KW20 (Hi KW20, gi: AAC22926), H. influenzae R2846 (Hi 12, gi: ADO96209), H. influenzae R2866 (Hi Int1, gi: ADO80811), H. influenzae 86-028NP (Hi 86-028NP, gi: AAX88650), N. meningitidis Z2491 (Nm Z2491, gi: CAM08236), Pasteurella multocida Pm70 (Pm 70, gi: AAK02817), and N. gonorrhoeae FA1090 (Ng FA1090, gi: AAW89132). Each ORF was amplified by PCR by Q5 high fidelity polymerase (New England Biolabs, M0494S) according to manufacturer recommendations with primers that contained 25-bp homology to pZE21-derived expression vector at the 5′ ends (Table S2) (15, 37). Q5 high fidelity polymerase was also used to generate linear, modified pZE21 vector for cloning by inverse PCR (Table S2). All DNA amplicons were verified for correct size and purified by agarose gel extraction (New England Biolabs, T1020S) from a 0.7% agarose gel. Each insert was cloned into the modified pZE21 backbone by NEBuilder HiFi assembly master mix (New England Biolabs, E2621S) according to manufacturer’s protocols. Reaction mixtures were incubated at 50°C for 15 min and transferred to ice. Chemically competent Es. coli DH10B cells were transformed by heat shock with 2 µL of each reaction, rescued in 1 mL of LB medium for 1 h at 37°C, and plated onto LB + KAN50 agar for overnight incubation at 37°C. Colony PCR (One*Taq* Quick-Load 2× master mix, New England Biolabs, M0486) (Table S2) and agarose gel electrophoresis were used to verify the presence of inserts, and sequence integrity was confirmed by Sanger sequencing (ACGT Inc., through the Northwestern Sanger Sequencing Core).

### Chloramphenicol reduction assay.

Chloramphenicol reduction by resting Es. coli cells expressing *nfsB* homologs was determined by a modification of the Merkel and Steers method (10). Four colonies of each Es. coli strain expressing *nfsB* genes from Hi KW20, Hi Int1, Hi 12, Hi 86-028NP, Nm Z2491, Ng FA1090, and Pm 70 or containing an empty vector were individually picked into 1 mL of LB + KAN50 medium in a sterile 1.5-mL tube. Cultures were incubated at 37°C with shaking overnight to reach stationary phase. The optical density of each culture was measured at 600 nm (OD_600_), and cultures were found to be uniform. Cultures were transferred onto ice and centrifuged at 4,000 relative centrifugal force (rcf) for 2 min to pellet cells. After supernatants were decanted, the cell pellets were resuspended on ice in 400 µL of 4°C MDG minimal medium (38) supplemented with chloramphenicol at 100 µg/mL. MDG medium was used to avoid the presence of compounds that can interfere with the Bratton-Marshall assay. MDG medium consists of “M” solution (25 mM Na_2_HPO_4_, 25 mM KH_2_PO_4_, 50 mM NH_4_Cl, and 5 mM Na_2_SO_4_), 0.4% glucose, 2 mM MgSO_4_, 0.2× trace metals solution (Teknova, T1001), and 50 µg/L leucine. To quench the t zero time point, cells were pelleted again as described above, and 100 µL of supernatant was removed from each sample directly into 100 µL of 20% trichloroacetic acid in water and held on ice. The cell pellets were resuspended into the remaining supernatant volume and incubated at 37°C. Additional quenches were performed as described above at 2.5 h and 5 h. After the final quench, each supernatant sample in 10% trichloroacetic acid was derivatized by the Bratton-Marshall method to detect aromatic amines (12, 15, 20) by the sequential addition of 25 µL of 0.1% sodium nitrite, 25 µL of 0.5% ammonium sulfamate, and 25 µL of 0.05% *N*-(1-naphthyl)ethylenediamine dihydrochloride (Bratton-Marshall reagent), with a 10-min room temperature incubation between each step. The relative quantity of aromatic amine was determined by measurement of the absorbance of each reaction at 550 nm on an Epoch Microplate Spectrophotometer (Biotek Instruments). The time series data were used to determine the apparent rate of chloramphenicol reduction in quadruplicate for each strain. Rates for each *nfsB*-expressing strain were compared to the empty vector control strain and tested for significance using Brown-Forsythe and Welch analysis of variance (ANOVA) tests with false-discovery rate correction by the method of Benjamini, Krieger, and Yekutieli, as implemented in GraphPad Prism 8.0 (GraphPad Software, La Jolla, CA, USA).

### Chloramphenicol susceptibility of Es. coli strains expressing *nfsB* homologs.

Four colonies each (*n* = 4 biological replicates) of Es. coli strains containing an empty pZE21 vector, pZE21 expressing chloramphenicol acetyltransferase (*cat*), or pZE21 expressing the Hi KW20 *nfsB* gene were inoculated into 1 mL of MH + KAN50 broth and incubated overnight at 37°C with shaking. Following growth, each culture was normalized to an OD_600_ of ∼1 absorbance unit (AU) by dilution in fresh MH + KAN50 broth and inoculated to an initial OD_600_ of 0.025 into 200 µL of MH + KAN50 broth supplemented with chloramphenicol, thiamphenicol, florfenicol, or Sch-24893 at 4 µg/mL in a 96-well flat-bottomed plate. The plate was sealed with a Breath-Easy membrane (Sigma, Z380059-1PAK) to prevent evaporation (39) and incubated with shaking at 37°C for 20 h. OD_600_ was measured by an Epoch plate reader following removal of the sealing membrane and manual resuspension of all wells. Blank readings and pathlength correction (1 cm) were taken on uninoculated wells with MH + KAN50 broth. Strain growth was determined by taking the average of four replicate results for each strain-amphenicol combination and evaluated using an ordinary two-way ANOVA with multiple comparison correction using the procedure of Benjamini, Krieger, and Yekutieli, as implemented in GraphPad Prism 8.0.

The chloramphenicol susceptibility assays below were performed twice in quadruplicate for each *nfsB* homolog-expressing strain of Es. coli (Hi KW20, Hi Int1, Hi 12, Hi 86-028NP, Nm Z2491, Ng FA1090, and Pm 70) for a total of *n* = 8 biological replicates. For each replicate, a single colony grown on LB + KAN50 agar was picked into MH + KAN50 liquid medium and incubated aerobically overnight at 37°C. The optical density of each culture was measured at 600 nm, and cultures were adjusted by addition of MH + KAN50 to give an initial density of between 0.75 and 1.25 absorbance units. These were used to inoculate MH + KAN50 cultures in a 96-well flat-bottomed plate with final volumes of 205 µL, each across a 12-step 2-fold dilution series of chloramphenicol ranging from 256 µg/mL to 0.125 µg/mL at an initial OD_600_ of between 0.02 and 0.05 absorbance units. The 96-well plates were sealed with a Breath-Easy membrane and incubated with shaking at 37°C for 20 h to 24 h. To read culture densities, the sealing membrane was removed to allow for manual resuspension of cell clumps prior to absorbance measurement at 600 nm on an Epoch microplate reader. Blank readings and pathlength correction (1 cm) were taken on control wells and applied to each plate prior to plotting final optical density against chloramphenicol concentration. The 50% inhibitory concentration (IC_50_) for each individual replicate was calculated by plotting the 4-factor sigmoidal function with a baseline of 0 using GraphPad Prism 8.0. Results for Es. coli expressing wild-type *nfsB* genes were evaluated for significance by comparison to empty vector controls using a Brown-Forsythe and Welch ANOVA test with Benjamini, Krieger, and Yekutieli false-discovery correction for multiple comparison in GraphPad Prism 8.0.

### Large-scale protein expression and purification.

Highly purified NfsB enzymes were prepared following the protocols established under the Midwest Center for Structural Genomics (MCSG) and the Center for Structural Genomics of Infectious Disease (CSGID) (40). NAD(P)H-flavin oxidoreductases from H. influenzae Rd KW20 (Hi KW20, gi: AAC22926), H. influenzae R2846 (Hi 12, gi: ADO96209), H. influenzae R2866 (Hi Int1, gi: ADO80811), H. influenzae 86-028NP (Hi 86-028NP, gi: AAX88650), N. meningitidis Z2491 (Nm Z2491, gi: CAM08236), Pasteurella multocida Pm70 (Pm 70, gi: AAK02817), and N. gonorrhoeae FA1090 (Ng FA1090, gi: AAW89132) were amplified from genomic DNA by KOD Hot Start DNA polymerase (Sigma-Aldrich, 71086), treated with T4 polymerase in the presence of dCTP, and cloned into the pMCSG53 vector and overexpressed in Es. coli BL21(DE3)-Gold (41). The pMCSG53 vector contains an N-terminal His_6_ tag with a tobacco etch virus (TEV) nuclear-inclusion-a endopeptidase (EC 3.4.22.44) protease cleavage site fused with the target protein. To test enzyme stability and solubility, a single colony for each homolog was picked into LB and induced with isopropyl-β-d-thiogalactoside (IPTG) overnight at 18°C. The cell lysates were analyzed for the presence of expressed proteins with the correct molecular weights and for protein solubility by small-scale nickel-nitrilotriacetic acid (Ni-NTA) affinity purification and TEV protease cleavage (following TEV cleavage, target proteins have three artificial residues [Ser, Asn, and Ala] on the N-terminal end). The P. multocida and N. gonorrhoeae enzymes were found to be insoluble, and their large-scale expression was not pursued.

For large-scale expression, cells were grown in 2-L plastic bottles in enriched M9 medium for selenomethionine (SeMet)-labeled proteins (42). Cell cultures in M9 medium were shaken at a rate of 180 rpm to an OD_600_ of ∼0.9 followed by incubation for 60 min at 4°C. Per liter of culture, 20 mL of l-SeMet with inhibitory amino acids (IAAC) solution was added followed by 0.5 mM IPTG after 20 min. After induction, all cell cultures were incubated overnight at 180 rpm at 18°C. The harvested cells were spun down at 7,000 rcf (Sorval Evolution RC centrifuge, Thermo Scientific) and resuspended in 25 mL of lysis buffer (50 mM HEPES pH 8.0, 500 mM NaCl, 20 mM imidazole, 10 mM β-mercaptoethanol, and 5% [vol/vol] glycerol) with protease inhibitor cocktail (Complete, Sigma). Lysozyme (Sigma) was added at a concentration of 1 mg/mL just before cells were stored at −80°C.

For purification, thawed cells were sonicated, and lysate was clarified by centrifugation at 28,000 rcf (Sorval Evolution RC centrifuge, Thermo Scientific) for 80 min, followed by filtration through a 0.45-µm filter (Millipore). Proteins were purified by Ni-immobilized metal affinity chromatography (IMAC-I) using a 5-mL HiTrap Chelating HP column charged with Ni^+2^ using an AKTA Express System (Cytiva). Since the recombinant TEV protease carried a noncleavable His_6_ tag (43), a second IMAC column was used to additionally purify the proteins by removal of TEV protease, cleaved His_6_ tag peptides, and any uncut recombinant protein.

The purified proteins were buffer exchanged into 20 mM Tris pH 7.5, 40 mM KCl, 0.1 mM FMN, and 1 mM tris(2-carboxyethyl) phosphine (TCEP; Amresco, Inc.) buffer and concentrated using an Amicon Ultra-15 centrifugal filter device (Millipore). The final concentrations were 67.9 mg/mL for Hi KW20, 123.6 mg/mL for Hi 12, 72.3 mg/mL for Hi Int1, 67.1 mg mL for Hi 86-028NP, and 51.1 mg/mL for Nm Z2491.

### *In vitro* analysis of chloramphenicol reduction products.

Prior to *in vitro* characterization, all soluble protein stocks (Hi KW20, Hi Int1, Hi 12, Hi 86-028NP, and Nm Z2491) were diluted 100-fold in 50 mM Tris pH 8 buffer and requantified by bicinchoninic acid (BCA) assay (Pierce, 23225) against a bovine serum albumin standard. Enzyme aliquots were normalized to an initial concentration of 0.2 µM based on the BCA assay values. To standardize reaction conditions, reduction of chloramphenicol to amino-chloramphenicol using NADPH was monitored by Bratton-Marshall assay. Soluble enzymes at 0.2 µM were each added to eight wells of a 96-well plate in 50-µL aliquots. To each well was added 50 µL of 500 µM (each) chloramphenicol and NADPH in 50 mM Tris. One reaction well for each enzyme was quenched with trichloroacetic acid approximately every minute for 7 min and analyzed by Bratton-Marshall derivatization as described above to generate a reaction product curve for the formation of amino-chloramphenicol.

To characterize the final product of chloramphenicol reduction, a simplified *in vitro* reaction was set up for each enzyme consisting of 4 mM NADPH, 1 mM chloramphenicol, and 1 μM enzyme in a 300-μL final volume. The usual components of buffer and saturating FMN were omitted to avoid potential matrix effects during LCMS analysis. Reactions were initiated by addition of enzyme and were incubated at room temperature (∼23°C) for 1 h before they were quenched by addition of 250 μL of ice-cold acetonitrile. Additional reactions with variable NADPH concentrations using only the Hi 12 NfsB enzyme were prepared as described above with NADPH at 0 mM, 1 mM, 2 mM, or 3 mM and allowed to incubate for 20 min before they were quenched. Two control reactions were performed as follows: one reaction with only enzyme omitted, and one reaction with enzyme omitted and with amino-chloramphenicol spiked in at a final concentration of 1 mM.

Quenched reactions were filtered through 0.45-μm high-performance liquid chromatography (HPLC) filter vials (Thomson Instrument Company, 35540-200) and analyzed by LCMS/MS using an Agilent 1290 Infinity II ultra-high-performance liquid chromatograph (UHPLC) coupled with a Q-Exactive mass spectrometer (Thermo Fisher Scientific). Reversed-phase (RP) chromatography was performed with water and acetonitrile as mobile phases at a flow rate of 700 µL min^−1^ on a Phenomenex Kinetex C_18_ RP-HPLC column (50 mm × 2.1 mm inner diameter, 1.3-µm particle size, 100-Å pore size) fitted with a Phenomenex SecurityGuard Ultra UHPLC guard cartridge. The 5-min UHPLC method consisted of an isocratic flow of 100% water for 0.25 min, a gradient of 0% to 45% aqueous acetonitrile for 3.95 min, a gradient of 45% to 100% aqueous acetonitrile for 0.20 min, and an isocratic flow of 100% acetonitrile for 0.1 min. Mass spectral data were acquired using a 200 to 900 *m/z* scan range at a resolution of 35,000, a maximum inject time of 200 ms, and an automatic gain control (AGC) target value of 1 × 10^6^. Tandem MS data were collected at a resolution of 17,500, a maximum inject time of 250 ms, an AGC target value of 5 × 10^6^, and stepped higher-energy collisional dissociation (HCD) energies of 20, 30, and 40 eV. The heated electrospray ionization source (HESI) was optimized to a spray voltage of 3.5 kV, a vaporizer temperature of 250°C, sheath gas pressure of 60 lb/in^2^, and auxiliary gas pressure of 15 lb/in^2^ to mitigate chloramphenicol in-source gas-phase reactions. Parallel reaction monitoring with a 2.0 *m/z* isolation window and an ion *m/z* inclusion list ensured that chloramphenicol-related ions were targeted for fragmentation. Data were analyzed using Thermo Xcalibur Qual Browser software version 4.0.27.10.

### *In vitro* characterization of enzymatic activity of NfsB homologs.

The Michaelis-Menten kinetics of chloramphenicol reduction by NADPH was performed as previously described (15). Briefly, quadruplicate 100-µL reactions made up of 50 mM Tris buffer pH 8, 1 mM NADPH, 1 µM FMN, and chloramphenicol concentrations from 2 mM to 31.25 µM and 0 µM were initiated by addition of enzyme to a final concentration of 0.1 µM. NADPH oxidation was followed by spectrometry at 340 nm with a 24-s interval using a BioTek Epoch spectrophotometer plate reader at ambient temperature (∼23°C). Slopes of the linear stage of each reaction progress curve were converted into rates using the NADPH extinction coefficient *ε*_340_ = 6,220 M^−1 ^cm^−1^ (31). Rates were plotted against chloramphenicol concentration to fit Michaelis-Menten kinetics curves to calculate apparent *k*_cat_ and *K_m_* using GraphPad Prism 8.0 using the following equation (*Et* represents the enzyme concentration 0.1 µM, and *X* represents the chloramphenicol concentration in µM):

$$Y=\mathrm{Et}\times k_{\text{cat}}\times \frac{X}{K_{m}+X}$$

Curves fit to all four replicates were plotted, and each replicate was analyzed individually to calculate mean and error statistics for *k*_cat_, *K_m_*, and *k*_cat_/*K_m_*, which are found in Table 1.

Determination of NADPH oxidation kinetics for each enzyme was performed as described above. Reactions were performed in quadruplicate in 100-µL volumes that contained 50 mM Tris buffer pH 8, 1 mM chloramphenicol, 1 µM FMN, and NADPH in concentrations from 500 µM to 31.25 µM and 0 µM. After initiation by addition of enzyme to a final concentration of 0.1 µM, NADPH oxidation was monitored as above. Initial reaction slopes were converted to rates and plotted to determine *k*_cat_, *K_m_*, and *k*_cat_/*K_m_* as above and are found in Table 1.

### NfsB enzyme crystallization, data collection, and structure determination.

Crystallization experiments for NfsB SeMet-labeled proteins of Hi KW20, Hi 12, Hi Int1, Hi 86-028NP, and Nm Z2491 were performed by the sitting drop vapor-diffusion method with the help of a Mosquito liquid handler instrument (TTP Labtech) in 96-well CrystalQuick plates (Greiner, Bio-one). Briefly, 0.4 μL of purified protein was mixed with 0.4 μL of crystallization solution and equilibrated over 135 μL of INDEX (Hampton Research), MCSG1, and MCSG4 (Anatrace), and PEGsII Suit (Nextal) well screening solutions. The final protein concentrations used in crystallization droplets were 11.3, 20.5, 12.1, 11.2, and 8.5 mg/mL for Hi KW20, Hi 12, Hi Int1, Hi 86-028NP, and Nm Z2491, respectively. The protein-to-ligand ratio in cocrystallization experiments was approximately 1:30. All crystallizations were performed at 16°C. Crystals suitable for structure determination appeared within a day and continued to appear under different crystallization conditions over the course of 3 to 4 weeks. For Hi 12 NfsB in complex with acetate, the best crystals grew under the MCSG1 D10 condition (0.2 M calcium acetate, 0.1 M sodium cacodylate pH 6.5, 40% polyethylene glycol [PEG] 300); for the Hi 12 NfsB and 4-nitrophenol complex, the best condition was MCSG1 E3 (0.05 M magnesium chloride, 0.1 M HEPES pH 7.5, 30% polyethylene glycol monomethyl ethers (PEGMME) 550); for Hi KW20 NfsB, the best condition was MCSG 1 C1 (0.1 M ammonium acetate, 0.1 M Bis-Tris HCl pH 5.5, 17% [wt/vol] PEG 10000/0.2 M calcium acetate, 0.1 M Tris-HCl pH 7.0, 20% [wt/vol] PEG 3000); for Hi Int1 NfsB, the best condition was MCSG1 C5 (0.2 M magnesium acetate and 20% [wt/vol] PEG 3350); for Hi 86-028NP NfsB, the best condition was MCSG1 E9 (0.2 M calcium chloride, 20% [wt/vol] PEG 3350); and for Nm Z2491 NfsB in complex with nicotinic acid, the best condition was MCSG1 C7 (0.2 M calcium chloride, 0.1 M Tris pH 8.5, and 25% [wt/vol] PEG 4000).

Before data collection, crystals in mother liquor plus typically 25% ethylene glycol were flash-frozen in liquid nitrogen. The crystal diffractions were measured using a Pilatus3 X 6M detector (SBC19-ID) or ADSC Q315r detector (SBC 19-BM) at a temperature of 100 K at the Structural Biology Center 19-ID or 19-BM beam line of the Advanced Photon Source (Argonne National Laboratory, Illinois). The diffraction data were indexed, integrated, and scaled using HKL3000 suite (44).

The structures were solved by single-wavelength anomalous dispersion (SAD) using shelxc/d/e (45) to find selenium sites, mlphare (46) for phasing, dm (47) to improve phases, and buccaneer/HKL builder (48) to autobuild the model, all implemented in the HKL3000 software package (44). The initial model was manually adjusted using COOT (49) and refined using COOT, PHENIX (50), and REFMAC (51) software packages. CC_1/2_ and Ramachandran favored/outlier percentages were determined by standard methods (52, 53). Throughout the structure refinements, the same 5% of reflections were kept out from the refinement.

The protein chain contains the N-terminal three-residue cloning artifact mentioned above (residues −2 to 0; Ser-Asn-Ala), which were disordered in most of these structures. In all structures, each protein molecule contains a well-ordered FMN molecule, a cofactor, in the corresponding active site. The final structure of Hi 12 NfsB converged to *R*_work_ and *R*_free_ of 0.142 and 0.159, respectively, with a resolution of 1.15 Å and contains 221 residues, including the N-terminal artifact residues, an FMN molecule, an acetic acid molecule, Ca and Cl ions, a formic acid molecule, and 187 water molecules. The final 1.45-Å structure of the Hi 12 NfsB.4-nitrophenol complex has an *R*_work_/*R*_free_ of 0.148/0.190 and includes the protein dimer, with chain A consisting of 223 residues (−2 to 220) and chain B consisting of 218 residues (3 to 220) missing five residues (including three purification artifact residues) at the N terminus. In addition, a substrate molecule (4-nitrophenol) and an FMN per protein chain, two ethylene glycols, a HEPES (buffer) molecule, a formic acid molecule, and 263 water molecules were found. The refinement of the structure of Hi KW20 NfsB against the 1.97-Å resolution data was converged to an *R*_work_ of 0.184 and an *R*_free_ of 0.217, and the structure comprises two protein chains with residues 2 to 220 and 3 to 220. In addition to the cofactor molecule FMN for each protein chain, an acetic acid and a chloride ion and 112 water molecules were also identified in the structure. For the Hi Int1 NfsB, the structure converged to an *R*_work_/*R*_free_ of 0.171/0.216 at a resolution of 1.95 Å and consists of the dimer of two 220-residue protein molecules (1 to 220). The structure also includes an FMN/protein chain, two acetic acids, two ethylene glycols, a sulfate, and 171 water molecules. The structure of Hi 86-028NP NfsB has a resolution of 1.68 Å with an *R*_work_/*R*_free_ of 0.152/0.191 and contains two dimers of four protein molecules with residues of 2 to 220, −2 to 220, 1 to 220, and −1 to 220 for the molecules A, B, C, and D, respectively, as well as an FMN for each chain, three ethylene glycols, two potassium, two calcium, and three chloride ions, and 358 water molecules. The structure of the NfsB homolog from Nm Z2491 was refined to *R*_work_ and *R*_free_ to 0.192 and 0.218 against the 1.75-Å data and consists of four protein molecules of 221 residues (1 to 221). Additionally, two ethylene glycols, four chloride ions, and 424 water molecules were found in the structure, and each protein chain contains an FMN and a nicotinic acid. The stereochemistry of the structures was checked with PROCHECK (54) and the Ramachandran plot and validated with the Protein Data Bank (PDB) validation server (55). The data collection and processing and the structure refinement statistics are presented in Table S3.

### Data availability.

The atomic coordinates of the NfsB structures from Hi 12, Hi 12 complexed with 4-nitrophenol, Hi 86-028NP, Hi Int1, Hi KW20, and Nm Z2491 have been deposited in the PDB with the accession IDs 7LDQ, 7RZL, 7S14, 7RZP, 7S1A, and 6WT2.
