# Supplementary material for: Functional and Structural Characterization of Diverse NfsB Chloramphenicol Reductase Enzymes from Human Pathogens
Source: Microbiol Spectr. 2022 Feb 23;10(2):e00139-22. doi: 10.1128/spectrum.00139-22 (PMC8941942; doi:10.1128/spectrum.00139-22)
Supplement: SUPPLEMENTAL FILE 1 — Supplemental material. Download SupplementalMaterials_Spectrum00139-22.pdf, PDF file, 1.9 MB [file supplementalmaterials_spectrum00139-22.pdf]

SUPPLEMENTAL FIGURE AND TABLE LEGENDS

Supplemental Table 1. Percent amino acid identity of investigated NfsB enzymes

Supplemental Table 2. Amplification primers

Supplemental Table 3. Crystallographic data and refinement statistics for NfsB structures

Supplemental Table 4. Chloramphenicol reduction citations for Figure 6

Supplemental Figure 1. NMR characterization of Sch-24893

a) ^{1}H-NMR and b) ^{13}C-NMR spectra of Sch-24893 dissolved in DMSO-d_6

Supplemental Figure 2. Representative chloramphenicol concentration-response curves for *E. coli* expressing *nfsB* homologs

Quadruplicate (n=4) concentration-response curves for *E. coli* strains expressing a) *H. influenzae nfsB* homologs, or b) *Neisseria* sp. and *P. multocida nfsB* homologs. Data points are averages of biological replicates (n=4) with standard deviation error bars. c) Chloramphenicol minimal inhibitory concentrations of n=8 replicates for *E. coli* strains expressing the indicated *nfsB* homolog.

Supplemental Figure 3. Chloramphenicol and amino-chloramphenicol dehydration product ions

a) MS^1 extracted ion chromatograms for chloramphenicol ($[M+H]^+$ *m/z* 323.0196) and its gas phase dehydration product ions ($[M+H]^+$ *m/z* 305.0093; $[M+H]^+$ *m/z* 274.9986). b) MS^1 extracted ion chromatograms for amino-chloramphenicol ($[M+Na]^+$ *m/z* 315.0274) and its gas phase dehydration product ions ($[M+H]^+$ *m/z* 275.0349 and $[M+H]^+$ *m/z* 257.0243).

Supplemental Figure 4. Bratton-Marshall *in vitro* chloramphenicol nitro reduction assay

Chloramphenicol reductase reactions were prepared and quenched at the indicated time points. Quenched reactions were analyzed for aromatic amine production by Bratton-Marshall derivatization with optical measurement at 550 nm to give enzyme reaction progress curves.

Supplemental Figure 5. Multiple sequence alignment of active NfsB enzymes

Multiple sequence alignment of the six chloramphenicol-reducing NfsB homologs and *P. multocida* Pm70 NfsB. Hi 12 NfsB secondary structural features are noted above the alignment. Fully conserved positions are shaded in red.

a)

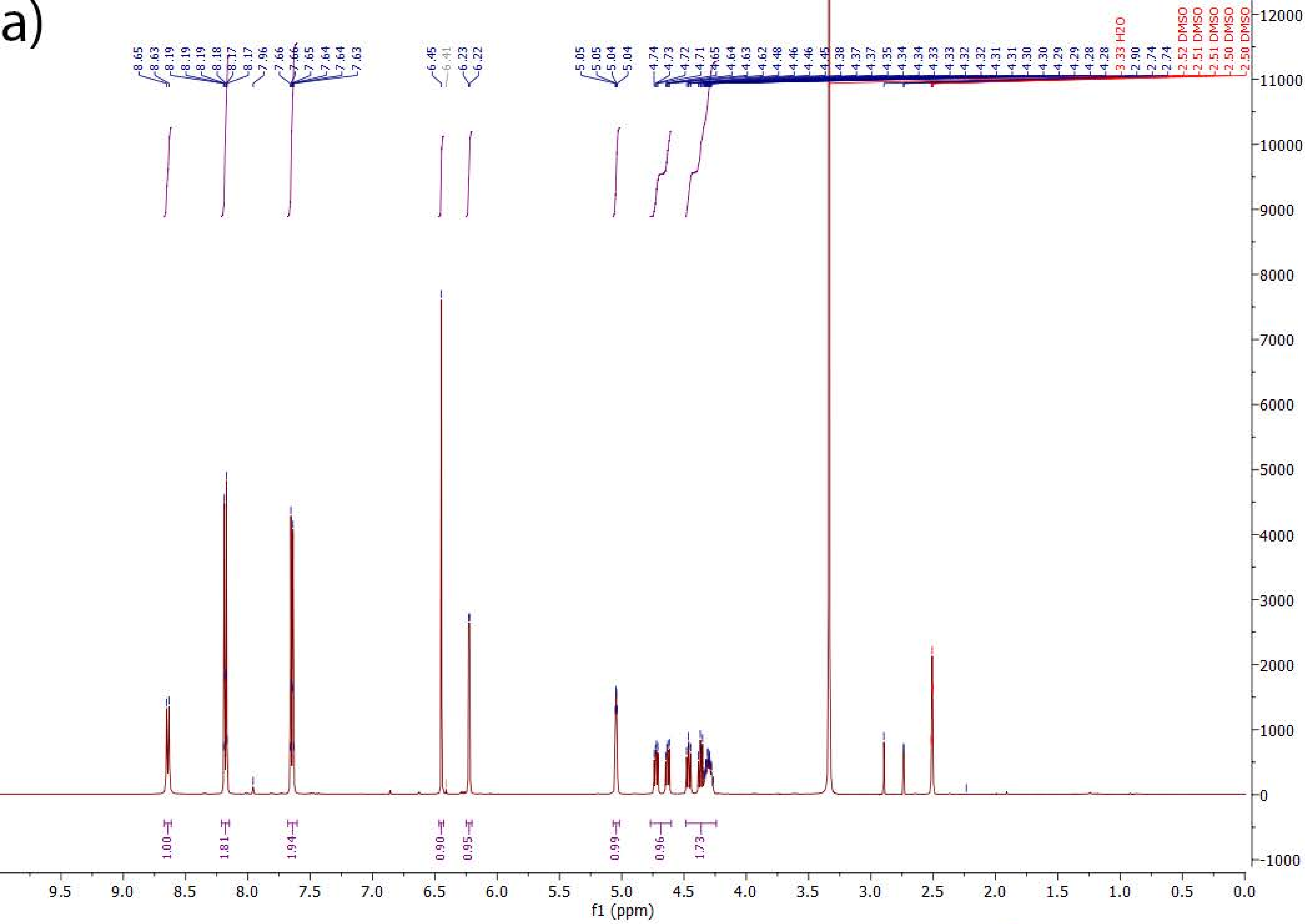

8.65
8.63
8.19
8.19
8.19
8.18
8.17
8.17
7.96
7.66
7.66
7.65
7.64
7.64
7.63
6.45
6.41
6.23
6.22
5.05
5.05
5.04
5.04
4.74
4.73
4.72
4.71
4.65
4.64
4.63
4.62
4.48
4.46
4.46
4.45
4.38
4.37
4.37
4.35
4.34
4.34
4.33
4.33
4.32
4.32
4.31
4.31
4.30
4.29
4.29
4.28
3.33 H2O
2.90
2.74
2.74
2.52 DMSO
2.51 DMSO
2.51 DMSO
2.50 DMSO
2.50 DMSO
1.00
1.81
1.94
0.90
0.95
0.99
0.96
1.73
f1 (ppm)

b)

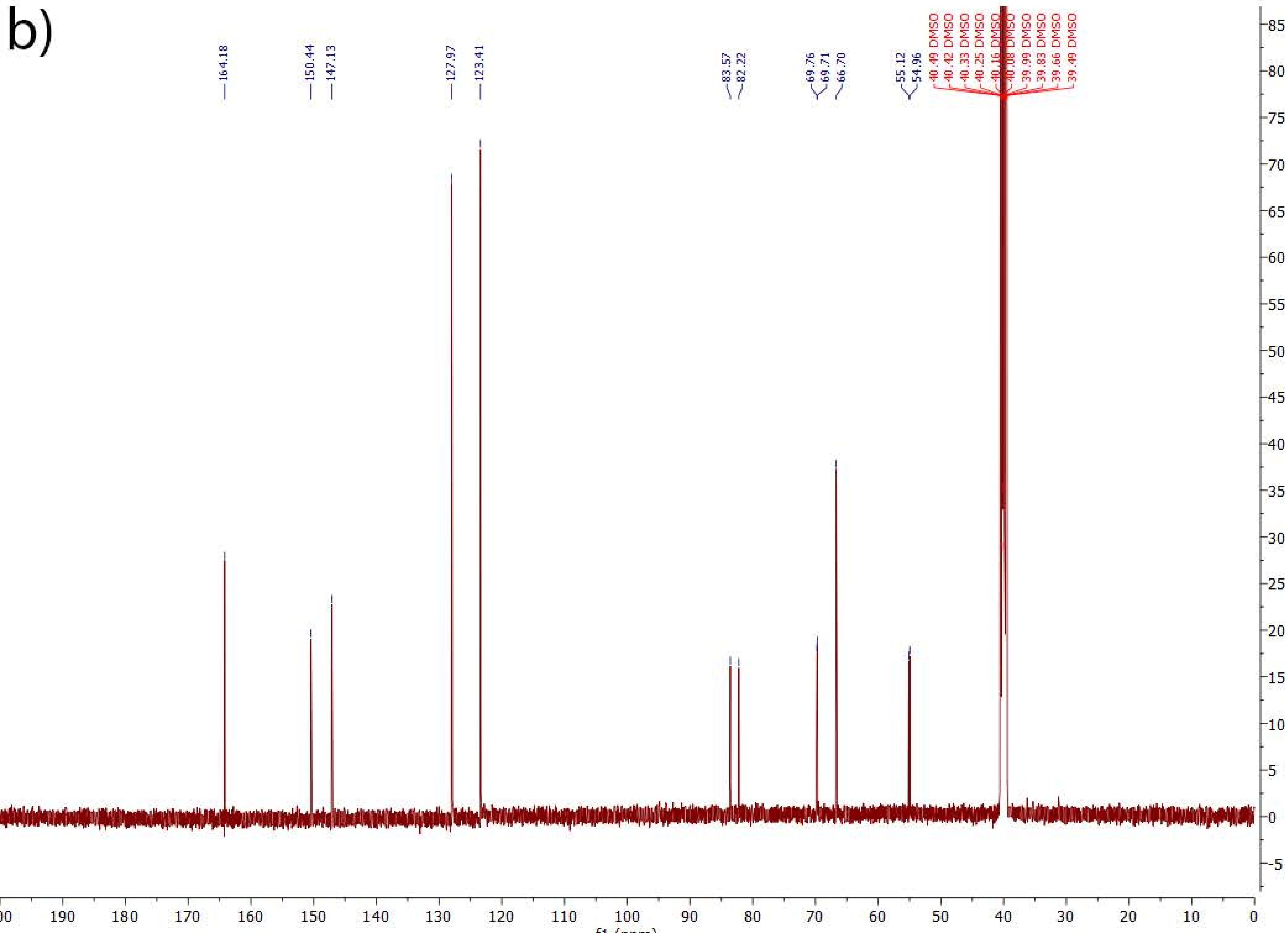

164.18
150.44
147.13
127.97
123.41
83.57
82.22
69.76
69.71
66.70
55.12
54.96
40.49 DMSO
40.42 DMSO
40.33 DMSO
40.25 DMSO
40.16 DMSO
40.08 DMSO
39.99 DMSO
39.83 DMSO
39.66 DMSO
39.49 DMSO

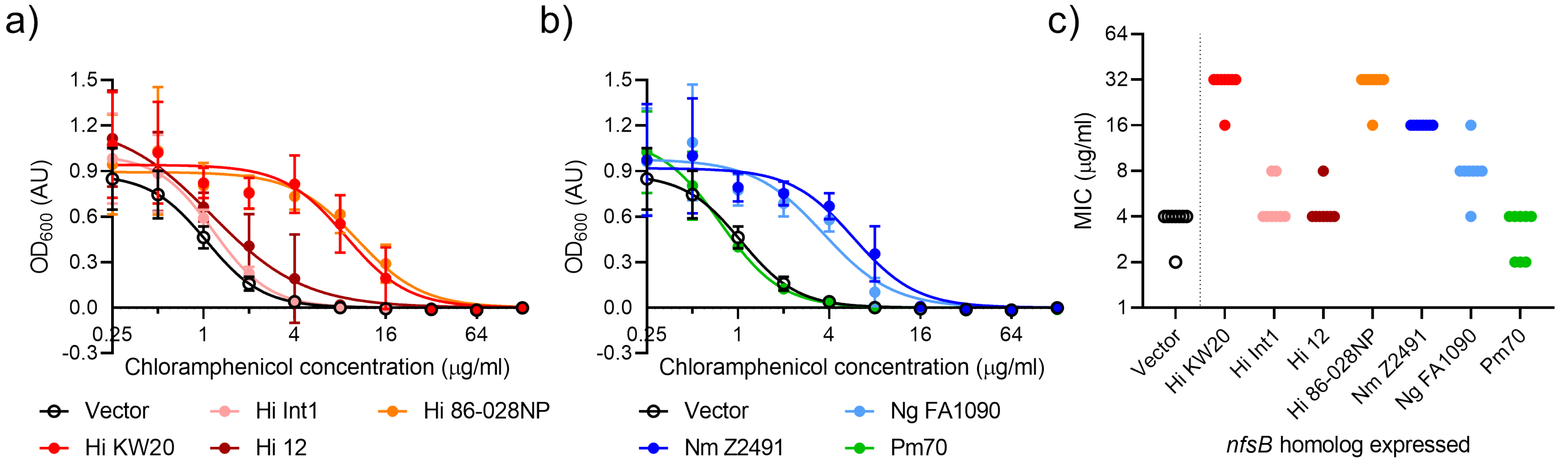

a)
1.5
1.2
0.9
0.6
0.3
0.0
-0.3
OD600 (AU)
0.25
1
4
16
64
Chloramphenicol concentration (μg/ml)
Vector
Hi KW20
Hi Int1
Hi 12
Hi 86-028NP
b)
Vector
Nm Z2491
Ng FA1090
Pm70
c)
64
32
16
8
4
2
1
MIC (μg/ml)
Vector
Hi KW20
Hi Int1
Hi 12
Hi 86-028NP
Nm Z2491
Ng FA1090
Pm70
nfsB homolog expressed

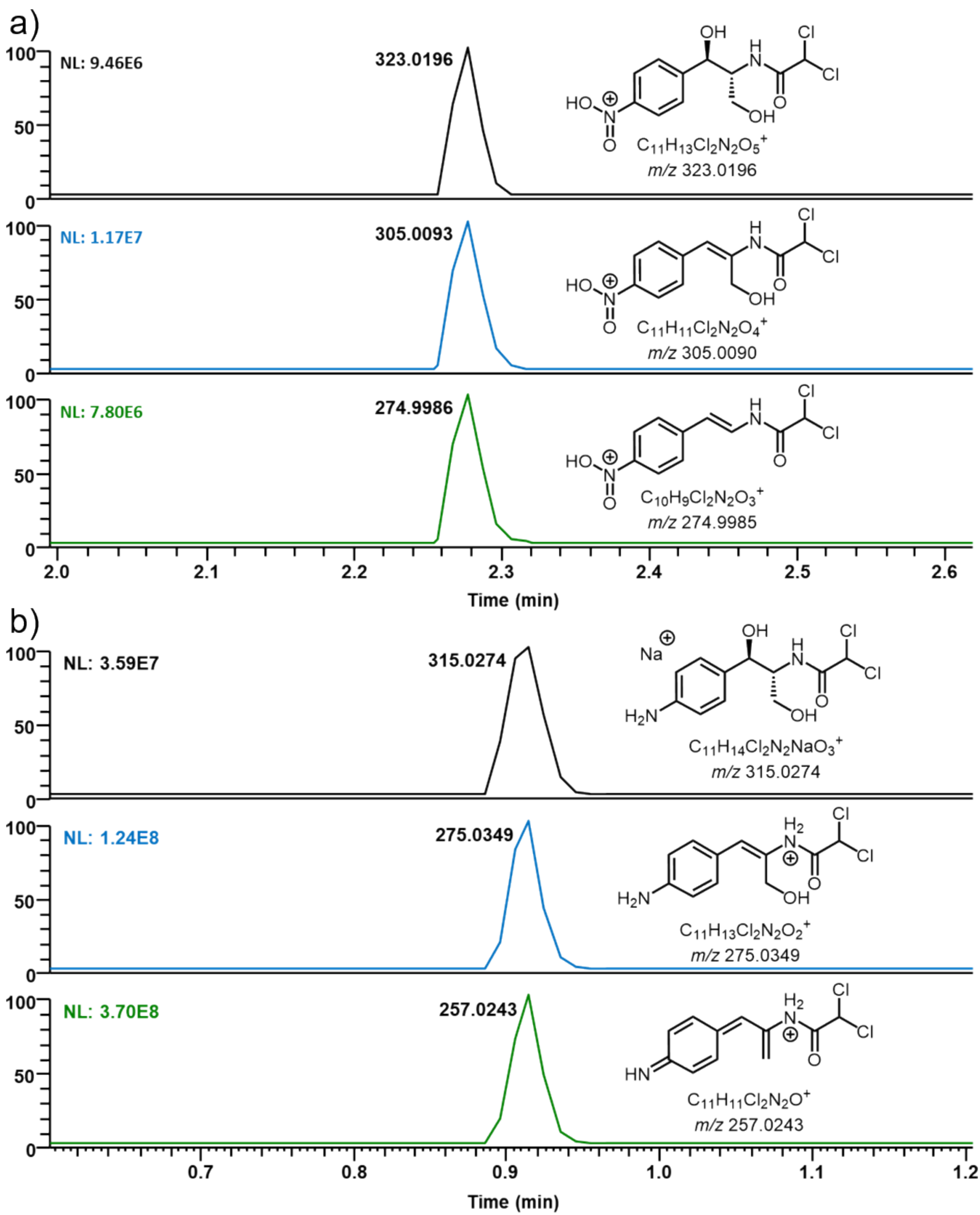

a)
NL: 9.46E6
323.0196
$C_{11}H_{13}Cl_2N_2O_5^+$
m/z 323.0196
NL: 1.17E7
305.0093
$C_{11}H_{11}Cl_2N_2O_4^+$
m/z 305.0090
NL: 7.80E6
274.9986
$C_{10}H_9Cl_2N_2O_3^+$
m/z 274.9985
Time (min)
b)
NL: 3.59E7
315.0274
$C_{11}H_{14}Cl_2N_2NaO_3^+$
m/z 315.0274
NL: 1.24E8
275.0349
$C_{11}H_{13}Cl_2N_2O_2^+$
m/z 275.0349
NL: 3.70E8
257.0243
$C_{11}H_{11}Cl_2N_2O^+$
m/z 257.0243
Time (min)

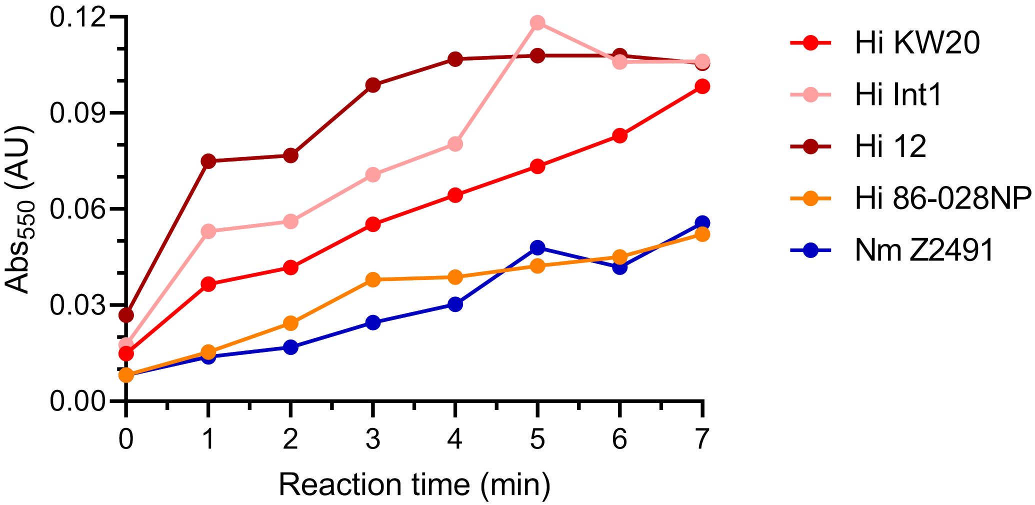

0.12
0.09
0.06
0.03
0.00
Abs550 (AU)
0
1
2
3
4
5
6
7
Reaction time (min)
Hi KW20
Hi Int1
Hi 12
Hi 86-028NP
Nm Z2491

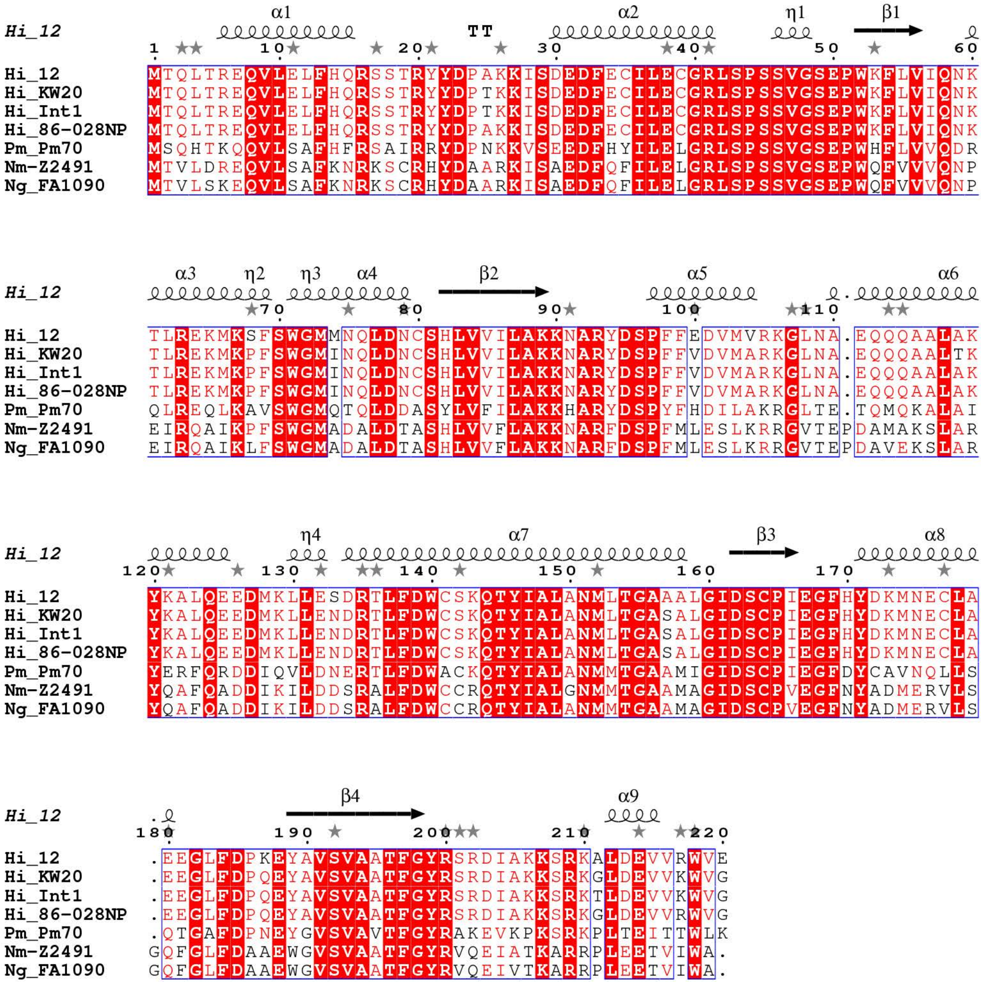
Hi_12
α1 TT α2 η1 β1
Hi_12 MTQLTREQVLELFHQRSSTRYYDPAKKISDEDFECILECGRLSPSSVGSEPWKFLVIQNK
Hi_KW20 MTQLTREQVLELFHQRSSTRYYDPTKKISDEDFECILECGRLSPSSVGSEPWKFLVIQNK
Hi_Int1 MTQLTREQVLELFHQRSSTRYYDPTKKISDEDFECILECGRLSPSSVGSEPWKFLVIQNK
Hi_86-028NP MTQLTREQVLELFHQRSSTRYYDPAKKISDEDFECILECGRLSPSSVGSEPWKFLVIQNK
Pm_Pm70 MSQHTKQQVLSAFHFRSAIRRYDPNKKVSEEDFHYILELGRLSPSSVGSEPWHFLVVQDR
Nm-Z2491 MTVLDREQVLSAFKNRKSCRHYDAARKISAEDFQFILELGRLSPSSVGSEPWQFVVVQNP
Ng_FA1090 MTVLSKEQVLSAFKNRKSCRHYDAARKISAEDFQFILELGRLSPSSVGSEPWQFVVVQNP
Hi_12
α3 η2 η3 α4 β2 α5 α6
Hi_12 TLREKMKSFSWGMMNQLDNCSHLVVILAKKNARYDSPFFEDVMVRKGLNA.EQQQAALAK
Hi_KW20 TLREKMKPFSWGMINQLDNCSHLVVILAKKNARYDSPFFVDVMARKGLNA.EQQQAALTK
Hi_Int1 TLREKMKPFSWGMINQLDNCSHLVVILAKKNARYDSPFFVDVMARKGLNA.EQQQAALAK
Hi_86-028NP TLREKMKPFSWGMINQLDNCSHLVVILAKKNARYDSPFFVDVMARKGLNA.EQQQAALAK
Pm_Pm70 QLREQLKAVSWGMQTQLDDASYLVFILAKKHARYDSPYFHDILAKRGLTE.TQMQKALAI
Nm-Z2491 EIRQAIKPFSWGMADALDTASHLVVFLAKKNARFDSPFMLESLKRRGVTEPDAMAKSLAR
Ng_FA1090 EIRQAIKLFSWGMADALDTASHLVVFLAKKNARFDSPFMLESLKRRGVTEPDAVEKSLAR
Hi_12
η4 α7 β3 α8
Hi_12 YKALQEEDMKLLESDRTLFDWCSKQTYIALANMLTGAAALGIDSCPIEGFHYDKMNECLA
Hi_KW20 YKALQEEDMKLLENDRTLFDWCSKQTYIALANMLTGASALGIDSCPIEGFHYDKMNECLA
Hi_Int1 YKALQEEDMKLLENDRTLFDWCSKQTYIALANMLTGASALGIDSCPIEGFHYDKMNECLA
Hi_86-028NP YKALQEEDMKLLENDRTLFDWCSKQTYIALANMLTGASALGIDSCPIEGFHYDKMNECLA
Pm_Pm70 YERFQRDDIQVLDNERTLFDWACKQTYIALANMMTGAAMIGIDSCPIEGFDYCAVNQLLS
Nm-Z2491 YQAFQADDIKILDDSRALFDWCCRQTYIALGNMMTGAAMAGIDSCPVEGFNYADMERVLS
Ng_FA1090 YQAFQADDIKILDDSRALFDWCCRQTYIALANMMTGAAMAGIDSCPVEGFNYADMERVLS
Hi_12
β4 α9
Hi_12 .EEGLFDPKEYAVSVAATFGYRSRDIAKKSRKALDEVVRWVE
Hi_KW20 .EEGLFDPQEYAVSVAATFGYRSRDIAKKSRKGLDEVVKWVG
Hi_Int1 .EEGLFDPQEYAVSVAATFGYRSRDIAKKSRKTLDEVVKWVG
Hi_86-028NP .EEGLFDPQEYAVSVAATFGYRSRDIAKKSRKGLDEVVRWVG
Pm_Pm70 .QTGAFDPNEYGVSVAVTFGYRAKEVKPKSRKPLTEITTWLK
Nm-Z2491 GQFGLFDAAEWGVSVAATFGYRVQEIATKARRPLEETVIWA.
Ng_FA1090 GQFGLFDAAEWGVSVAATFGYRVQEIVTKARRPLEETVIWA.

Supplemental table 1. Percent amino acid identity of investigated NfsB enzymes

Homolog	Hi KW20	Hi Int1	Hi 12	Hi 86-028NP	Nm Z2491	Ng FA1090
**Hi Int1**	99.09					
**Hi 12**	94.98	95.43				
**Hi 86-028NP**	98.64	98.64	96.35			
**Nm Z2491**	58.18	58.64	59.09	59.09		
**Ng FA1090**	56.82	57.27	58.18	57.73	96.83	
**Pm 70**	60.73	61.19	60.45	61.19	58.64	59.09

Supplemental table 2. Amplification primers

Primer name	Sequence	Use
49TSC	GTGCCCCCCCTCGAG	Inverse PCR amplification of pZE21 derivative pELLA
50TSC	GTGACCTTTCTCCTCTTTAATGAATTC	Inverse PCR amplification of pZE21 derivative pELLA
6426TSC	ATTCATTAAAGAGGAGAAAGGTCACATGACACAGTTGACCCG	Hi Int1 *nfsB* forward
6427TSC	ATTCATTAAAGAGGAGAAAGGTCACATGACCCAATTGACGC	Hi 12 *nfsB* forward
6428TSC	ATTCATTAAAGAGGAGAAAGGTCACATGACACAACTGACACG	Hi 86-028NP *nfsB* forward
6429TSC	ATTCATTAAAGAGGAGAAAGGTCACATGACTGTATTGGATCGCG	Nm Z2491 *nfsB* forward
6430TSC	ATTCATTAAAGAGGAGAAAGGTCACATGACAGTCCTTAGCAAAGA	Ng FA1090 *nfsB* forward
6431TSC	ATTCATTAAAGAGGAGAAAGGTCACATGTCTCAACACACGAAG	Pm Pm70 *nfsB* forward
6432TSC	TACCGTCGACCTCGAGGGGGGGCACTTAACCGACCCATTTAACCA	Hi Int1 *nfsB* reverse
6433TSC	TACCGTCGACCTCGAGGGGGGGCACTTACTCCACCCAGCGTA	Hi 12 *nfsB* reverse
6434TSC	TACCGTCGACCTCGAGGGGGGGCACTTAGCCCACCCAGC	Hi 86-028NP *nfsB* reverse
6435TSC	TACCGTCGACCTCGAGGGGGGGCACTTACGCCCAAATAACGGTTT	Nm Z2491 *nfsB* reverse
6436TSC	TACCGTCGACCTCGAGGGGGGGCACTTAGGCCCAGATCACG	Ng FA1090 *nfsB* reverse
6437TSC	TACCGTCGACCTCGAGGGGGGGCACTTACTTCAACCAGGTTGTGA	Pm70 *nfsB* reverse
6463TSC	CCAGTTTACTTTGCAGGGCTTCCCAACC	Amplification of pELLA cloning site in colony PCR
6464TSC	GCCTTTGAGTGAGCTGATACCGCTCG	Amplification of pELLA cloning site in colony PCR

Supplemental table 3: Crystallographic data and refinement statistics for NfsB structures

	**Hi 12**	**Hi 12 + 4-nitrophenol**	**Hi KW20**	**Hi** Int1	**Hi** 86-028NP	**Nm** Z2491
Wavelength, Å	0.97918	0.97890	0.97919	0.97919	0.97918	0.97951
X-ray source	SBC-19ID	SBC-19ID	SBC-19BM	SBC-19BM	SBC-19ID	SBC-19ID
Resolution limit[a], Å	1.15 (1.17-1.15)	1.45 (1.48-1.45)	1.97 (2.00-1.97)	1.95 (1.98-1.95)	1.68 (1.68-1.65)	1.75 (1.78-1.75)
Space group	C2	$P2_12_12_1$	$P3_1$	$P3_1$	$P2_1$	$P2_1$
Unit cell: *a, b, c,* (Å), *α, β, γ*, (°)	53.1,84.84, 53.98, *β*=115.8	54.73, 77.70, 90.62	*a*=*b*=56.82, *c*=122.92	*a*=*b*=56.17, *c*=122.49	56.83, 54.78, 134.45, *β*=90.04	81.56, 70.17, 85.62, *β*=89.96
# molecules in ASU	1	2	2	2	4	4
Unique reflections	74481 (3643)	68981 (3288)	31244 (1475)	31406 (1548)	97736 (4709)	96271
Multiplicity	4.6 (3.7)	11.4 (4.9)	7.2 (5.1)	5.0 (2.8)	4.4 (3.9)	5.0 (4.0)
Completeness, %	98.0 (95.8)	99.7 (95.9)	99.8 (97.0)	99.9 (99.5)	98.9 (96.4)	99.3 (98.9)
I/σI	18.8 (3.2)	25.8 (1.7)	22.4 (1.2)	13.5 (1.1)	11.9 (0.98)	11.3 (1.12)
Wilson B-factor, $Å^2$	12.9	12.2	35.9	29.7	23.0	22.0
R-merge[b]	0.101 (0.496)	0.098 (1.129)	0.111 (1.182)	0.158 (1.072)	0.105 (0.931)	0.188 (1.161)
$CC_{1/2}$ (highest resolution shell)[c]	0.811	0.547	0.394	0.361	0.453	0.616
R_{work}/R_{free}[d]	0.142/0.159	0.148/0.190	0.184/0.217	0.171/0.216	0.152/0.191	0.192/0.218
Protein residue ranges	A: 0-220	A: -2-220 B: 3-220	A: 2-220 B: 3-220	A, B: 1-220	A: 2-220 B: -2-220 C: 1-220 D: -1-219	A,B,C,D: 1-221
# Other atoms (water/the rest)	187/43	263/127	112/73	171/83	358/147	424/175
RMS bonds/angle	0.005/0.801	0.008/1.026	0.008/0.903	0.011/1.197	0.003/1.18	0.007/0.827
Ramachandran favored/outlier[e], %	97.17/0.0	97.68/0.46	97.46/0.23	96.56/0.0	95.23/0.58	94.57/0.85
Mean B-factor, $Å^2$ protein/ligand/water	17.2 16.1/15.8/29.6	17.3 16.6/19.51/26.35	48.9 49.2/42.8/44.4	36.6 36.6/33.3/38.0	29.3 31.6/23.9/30.4	28.5 28.3/25.2/32.6
PDBID	7LDQ	7RZL	7S1A	7RZP	7S14	6WT2

[a] Resolution ranges for the highest resolution shells and values for highest resolution shells in parentheses throughout the table.

[b] Rmerge = ΣhΣj|Ihj–<Ih>|/ΣhΣjIhj, where Ihj is the intensity of observation j of reflection h.

[c] As defined by Karplus and Diederichs (42)

[d] R = Σh|Fo|–|Fc|/Σh|Fo| for all reflections, where Fo and Fc are observed and calculated structure factors, respectively. R_{free} is calculated analogously for the test reflections, randomly selected and excluded from the refinement.

[e] As defined by Molprobity (43)

Supplemental table 4. Chloramphenicol reduction citations for figure 6

Taxa	Native host	Heterologous	*In vitro*
H. influenzae KW20	(12)	This manuscript, (15)	This manuscript, (15–17)
H. influenzae Int1	(12)	This manuscript	This manuscript
H. influenzae 86-028NP	(12)	This manuscript	This manuscript
H. influenzae 12	(12)	This manuscript	This manuscript
N. meningitidis Z2491	(12)	This manuscript	This manuscript
N. gonorrhoeae FA1090	(12)	This manuscript	
P. multocida Pm70	(12)	This manuscript	
S. enterica		(15)	
E. coli	(9)	(15)	
E. cloacae		(15)	
S. pyogenes	(56)	(15)	
C. acetobutylicum	(11)	(15)	
L. lactis			(21)
